# Destabilization of mRNAs enhances competence to initiate meiosis in mouse spermatogenic cells

**DOI:** 10.1242/dev.202740

**Published:** 2024-07-15

**Authors:** Natalie G. Pfaltzgraff, Bingrun Liu, Dirk G. de Rooij, David C. Page, Maria M. Mikedis

**Affiliations:** ^1^ Reproductive Sciences Center, Division of Developmental Biology, Cincinnati Children's Hospital Medical Center, Cincinnati, OH 45229, USA; ^2^ Whitehead Institute, Cambridge, MA 02142, USA; ^3^ Howard Hughes Medical Institute, Whitehead Institute, Cambridge, MA 02142, USA; ^4^ Department of Biology, Massachusetts Institute of Technology, Cambridge, MA 02139, USA; ^5^ Department of Pediatrics, University of Cincinnati College of Medicine, Cincinnati, OH 45229, USA

**Keywords:** MEIOC, RBM46, YTHDC2, Germ cells, Spermatogenesis

## Abstract

The specialized cell cycle of meiosis transforms diploid germ cells into haploid gametes. In mammals, diploid spermatogenic cells acquire the competence to initiate meiosis in response to retinoic acid. Previous mouse studies revealed that MEIOC interacts with RNA-binding proteins YTHDC2 and RBM46 to repress mitotic genes and to promote robust meiotic gene expression in spermatogenic cells that have initiated meiosis. Here, we have used the enhanced resolution of scRNA-seq and bulk RNA-seq of developmentally synchronized spermatogenesis to define how MEIOC molecularly supports early meiosis in spermatogenic cells. We demonstrate that MEIOC mediates transcriptomic changes before meiotic initiation, earlier than previously appreciated. MEIOC, acting with YTHDC2 and RBM46, destabilizes its mRNA targets, including the transcriptional repressors *E2f6* and *Mga*, in mitotic spermatogonia. MEIOC thereby derepresses E2F6- and MGA-repressed genes, including *Meiosin* and other meiosis-associated genes. This confers on spermatogenic cells the molecular competence to, in response to retinoic acid, fully activate the transcriptional regulator STRA8-MEIOSIN, which is required for the meiotic G1/S phase transition and for meiotic gene expression. We conclude that, in mice, mRNA decay mediated by MEIOC-YTHDC2-RBM46 enhances the competence of spermatogenic cells to initiate meiosis.

## Teaser

**Summary:** The RNA-binding complex MEIOC-YTHDC2-RBM46 destabilizes its mRNA targets, including transcriptional repressors. This activity facilitates the retinoic acid-dependent activation of *Meiosin* gene expression and transition into meiosis.

## INTRODUCTION

Sexual reproduction depends on meiosis: a specialized cell cycle that produces haploid gametes via one round of DNA replication followed by two rounds of chromosome segregation. The major chromosomal events of meiosis, including pairing, synapsis and crossing over of homologous chromosomes, are generally conserved across eukaryotes. By contrast, the mechanisms that govern the transition from mitosis to meiosis are less well conserved (Kimble, 2011). For example, in budding yeast, meiotic initiation occurs when multiple inputs converge to activate a master transcription factor (IME1) that upregulates meiotic gene expression (Kassir et al., 1988; Smith et al., 1990; reviewed by van Werven and Amon, 2011). In *Drosophila melanogaster*, the transition from mitosis to meiosis occurs via translational repression mediated by the RNA helicase Bgcn and its binding partners Bam and Tut (Chen et al., 2014; Gonczy et al., 1997; Insco et al., 2009, 2012; McKearin and Spradling, 1990).

In mammals, transcriptional activation induced by extrinsic signaling plays a central role in meiotic initiation (i.e. the meiotic G1/S transition). During the transition from mitosis to meiosis (i.e. the ‘premeiotic’ stage during oogenesis and the ‘preleptotene’ stage during spermatogenesis), retinoic acid induces meiotic entry by transcriptionally activating *Stra8* and *Meiosin* (Anderson et al., 2008; Baltus et al., 2006; Dokshin et al., 2013; Ishiguro et al., 2020). *In vitro*, nutrient restriction synergizes with retinoic acid to induce meiotic entry in spermatogenic cells (Zhang et al., 2021). The STRA8-MEIOSIN heterodimer transcriptionally activates expression of G1/S cyclins and meiotic factors that orchestrate the chromosomal events of meiotic prophase I (Anderson et al., 2008; Baltus et al., 2006; Ishiguro et al., 2020; Kojima et al., 2019; Mark et al., 2008; Soh et al., 2015; Zhang et al., 2023). In mice lacking *Stra8* or *Meiosin* on a C57BL/6 genetic background, premeiotic oogonia and preleptotene spermatocytes halt their development and fail to progress to meiotic prophase I (Anderson et al., 2008; Baltus et al., 2006; Dokshin et al., 2013; Ishiguro et al., 2020). Indeed, *Stra8*-null premeiotic oogonia and preleptotene spermatocytes, as well as *Meiosin*-null premeiotic oogonia, fail to undergo meiotic DNA replication (Anderson et al., 2008; Baltus et al., 2006; Dokshin et al., 2013; Ishiguro et al., 2020). [*Meiosin*-null preleptotene spermatocytes initiate an S phase, but it remains unclear whether it is mitotic or meiotic in nature (Ishiguro et al., 2020).]

In the mammalian testis, competence to initiate meiosis in response to retinoic acid is acquired during spermatogenesis. In mitotic spermatogonia, DMRT1 postpones the acquisition of competence, thereby preventing precocious meiotic entry, by repressing retinoic acid-dependent transcription as well as *Stra8*, and likely *Meiosin*, gene expression (Matson et al., 2010; Ishiguro et al., 2020). As a result, undifferentiated spermatogonia exposed to endogenous retinoic acid express *Stra8* at low levels, but do not express *Meiosin*, and consequently become mitotic differentiating spermatogonia (Ishiguro et al., 2020; Matson et al., 2010). Differentiating spermatogonia prematurely exposed to exogenous retinoic acid may express STRA8 protein, but they do not initiate meiosis (Endo et al., 2015; Johnson et al., 2023). At the mitosis-to-meiosis transition, the SCF E3 ubiquitin ligase complex degrades DMRT1 (Matson et al., 2010; Nakagawa et al., 2017), thereby conferring upon spermatogenic cells the competence to express *Stra8* and *Meiosin*, and to initiate meiosis in response to retinoic acid. To date, the SCF complex is the only known positive regulator of spermatogenic cell competence to initiate meiosis.

Post-transcriptional regulation of mRNA also plays a role in governing the mitosis-to-meiosis transition in mammals. After meiotic initiation, MEIOC acts together with the RNA helicase YTHDC2 and the RNA-binding protein RBM46 to regulate progression through meiotic S phase into meiotic prophase I (Abby et al., 2016; Bailey et al., 2017; Hsu et al., 2017; Jain et al., 2018; Qian et al., 2022; Soh et al., 2017; Wojtas et al., 2017). The three proteins are required to increase meiotic gene expression and also to repress the mitotic cell cycle program, thereby inhibiting a premature and aberrant metaphase several days before wild-type meiotic metaphase I. MEIOC, YTHDC2 and RBM46 are homologs of *Drosophila* proteins Bam, Bgcn and Tut, respectively (Bailey et al., 2017; Jain et al., 2018; Peart et al., 2022; Qian et al., 2022; Soh et al., 2017). Like their homologs, MEIOC, YTHDC2 and RBM46 interact with one another, as well as with mRNA (Abby et al., 2016; Bailey et al., 2017; Hsu et al., 2017; Jain et al., 2018; Li et al., 2022; Peart et al., 2022; Qian et al., 2022; Saito et al., 2022; Soh et al., 2017). YTHDC2 also interacts with exoribonuclease XRN1, while RBM46 recruits nonsense-mediated decay protein UPF1 and subunits of the cytoplasmic deadenylase CCR4-NOT complex, which suggests that YTHDC2 and RBM46 mRNA targets are degraded (Kretschmer et al., 2018; Li et al., 2022; Qian et al., 2022; Wojtas et al., 2017). The mechanism by which the MEIOC-YTHDC2-RBM46 complex post-transcriptionally regulates its mRNA targets after meiotic initiation remains poorly defined.

Here, we molecularly dissect the activity of MEIOC in mouse spermatogenic cells during the mitosis-to-meiosis transition using two parallel approaches: (1) single-cell RNA sequencing (scRNA-seq) analysis of postnatal testes and (2) bulk RNA-seq analysis of developmentally synchronized testes with histologically verified staging. Both approaches reveal that *Meioc*-null germ cells developmentally diverge from wild-type spermatogenic cells at the meiotic G1/S transition, earlier than previously appreciated. Furthermore, we find that MEIOC activity leads to an increase in meiosis-associated gene expression in late mitotic spermatogonia, before meiotic initiation. We discover that MEIOC destabilizes mRNAs encoding transcriptional repressors of meiotic gene expression. This inhibition of repressors facilitates activation of the master meiotic transcriptional regulator STRA8-MEIOSIN in response to retinoic acid. Therefore, a post-transcriptional repressor of mRNA, acting in parallel to the degradation of DMRT1 by the SCF complex, enhances the competence of spermatogenic cells to activate the meiotic transcriptional regulator and initiate meiosis.

## RESULTS

### MEIOC drives a transcriptomic shift at the meiotic G1/S transition

To characterize the molecular consequences of MEIOC activity during spermatogenesis, we performed 10x Genomics Chromium-based scRNA-seq on *Meioc*-null and wild-type P15 testes, and identified germ cell clusters by cell type-enriched marker expression and transcriptome-based cell cycle analysis (Figs S1-S3, Table S2). These germ cell clusters, identified in both wild-type and *Meioc*-null testes, consisted of four clusters in mitosis [undifferentiated spermatogonia (Undiff), differentiating type A_1_-A_4_ spermatogonia (A1-4), differentiating intermediate and type B spermatogonia (In/B) and differentiating type B spermatogonia in G2/M phase (B G2M)]; three clusters spanning the mitosis-to-meiosis transition [preleptotene spermatocytes in G1, early S and late S phase (pL G1, pL eS and pL lS, respectively)]; and three clusters in meiotic prophase I [leptotene spermatocytes (L), zygotene spermatocytes (Z) and pachytene spermatocytes (P)]. Although previous histological characterization found that *Meioc*-null spermatogenic cells did not progress beyond early zygotene (Abby et al., 2016; Soh et al., 2017), some *Meioc*-null cells were designated as pachytene spermatocytes in the scRNA-seq clustering. This indicates that transcriptomic progression in *Meioc*-null spermatocytes is not reflected at the histological level. We also identified a cluster composed of *Meioc*-null spermatocytes, which we designated as ‘Mutant-only’ (Mut). As this cluster primarily comprised cells in G2/M phase (Fig. S1C), it likely includes the aberrant metaphase spermatogenic cells found in P15 *Meioc*-null testes, days before meiotic metaphase I occurs in the wild-type testis (Abby et al., 2016; Soh et al., 2017).

**Fig. 1.**
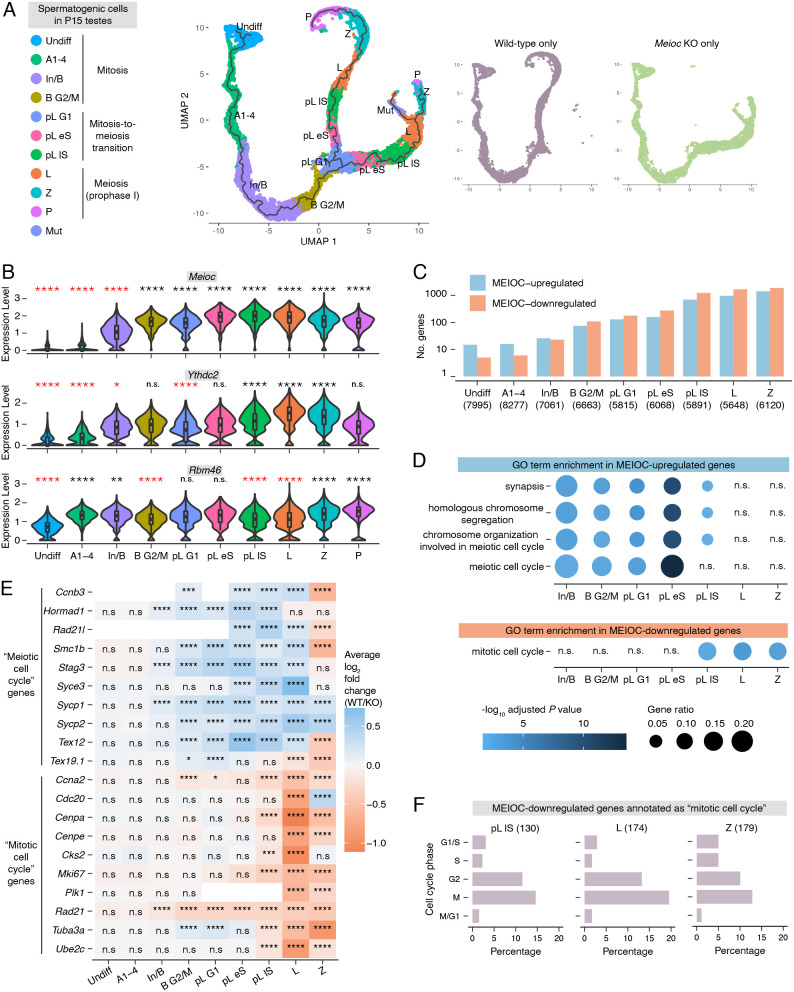
***Meioc*-null germ cells transcriptomically diverge from wild-type germ cells during the G1/S phase transition.** (A) UMAP visualization with pseudotime trajectory of wild-type and *Meioc* knockout germ cells from P15 testes. Large plot on the left displays both wild-type and *Meioc* knockout cells. Smaller plots on the right display cells of a single genotype. (B) Expression levels of *Meioc*, *Ythdc2* and *Rbm46* in germ cell clusters from wild-type testes. Black and red asterisks indicate enrichment or depletion, respectively, relative to all other germ cells. (C) Number of genes identified as MEIOC upregulated [log_2_ fold change (wild type/knockout)>0.1, *P*adj.<0.05] and downregulated [log_2_ fold change (wild type/knockout)<−0.1, *P*adj.<0.05] within each germ cell cluster. (D) Gene Ontology (GO) term enrichment analysis for MEIOC-upregulated and -downregulated genes shown. Graph for MEIOC-upregulated genes displays the top four enriched categories identified in the B G2/M cluster. Graph for MEIOC-downregulated genes displays a selected category of interest. (E) Heatmap of log_2_ fold changes for selected genes annotated as GO terms ‘meiotic cell cycle’ or ‘mitotic cell cycle’. (F) Associated cell cycle phases for MEIOC-downregulated genes that are annotated as GO term ‘mitotic cell cycle’. Only those genes whose expression is associated with a specific cell cycle phase are included. Cluster abbreviations: Undiff, undifferentiated spermatogonia; A1-4, differentiating type A1-A4 spermatogonia; In/B, differentiating intermediate and type B spermatogonia; B G2/M, differentiating type B spermatogonia in G2/M phase; pL G1, preleptotene spermatocytes in G1 phase; pL eS, preleptotene spermatocytes in early S; pL lS, preleptotene spermatocytes in late S phase; L, leptotene spermatocytes; Z, zygotene spermatocytes; P, pachytene spermatocytes; Mut, mutant spermatocytes. *Padj.<0.05; **Padj.<0.01; ***Padj.<0.001; ****Padj.<0.0001; n.s., not significant. See Table S1 for details on statistical testing.

We then applied pseudotime analysis to computationally reconstruct the developmental trajectory of the spermatogenic cells from undifferentiated spermatogonia to pachytene spermatocytes (Fig. 1A). *Meioc*-null germ cells followed the same trajectory as wild-type cells during the mitotic stages of spermatogenesis but diverged in the G1 phase of the preleptotene stage, immediately before meiotic S phase. As spermatogenic cells are classically staged via histology, we used histologically staged samples to independently verify these pseudotime results. We developmentally synchronized spermatogenesis to obtain *Meioc*-null and wild-type testes enriched for preleptotene spermatocytes (Fig. S4A). After histologically verifying staging (Fig. S4B), preleptotene-enriched testes were analyzed via bulk RNA-seq for differential expression, and we identified over 2000 transcripts as differentially abundant (Fig. S4C, Table S9). We then asked whether MEIOC impacts the G1/S transition in the preleptotene-enriched testes. We compared percentile ranks for fold changes (wild type/*Meioc* knockout) for genes enriched at specific cell cycle phases (see Materials and Methods). As a positive control for the disrupted G1/S transition, we re-analyzed a bulk RNA-seq dataset of preleptotene spermatocytes sorted from wild-type and *Stra8*-null testes on a C57BL/6 background (Kojima et al., 2019). This analysis revealed that, in preleptotene spermatocytes, MEIOC increased transcript abundance for genes associated with G1/S, S and G2, similar to STRA8 (Fig. S4D, Table S10). In total, both scRNA-seq and bulk RNA-seq datasets demonstrate that MEIOC drives a major transcriptomic shift in spermatogenic cells at the meiotic G1/S transition. This is earlier than previously appreciated, as previous histological studies reported that *Meioc*-null spermatogenic cells diverge from wild-type controls after entering meiotic S phase (Abby et al., 2016; Soh et al., 2017).

### MEIOC begins to affect the abundance of meiotic, rather than mitotic, transcripts before the meiotic G1/S transition

MEIOC protein and its binding partner YTHDC2 are first detected immunohistochemically at the preleptotene stage, but YTHDC2 protein is also detected via western blotting in mitotic spermatogonia (Abby et al., 2016; Bailey et al., 2017; Hsu et al., 2017; Jain et al., 2018; Soh et al., 2017; Wojtas et al., 2017). The binding partner RBM46 is detected via immunostaining in mitotic spermatogonia through meiotic spermatocytes (Peart et al., 2022; Qian et al., 2022). We found that *Meioc* and *Ythdc2* transcripts became abundant in late mitotic spermatogonia (In/B and B G2M clusters), whereas *Rbm46* was highly abundant in all germ cell clusters examined (Fig. 1B, Table S2). These observations raised the possibility that MEIOC, potentially in collaboration with YTHDC2 and RBM46, shapes the germline transcriptome in late mitotic spermatogonia, before the protein can be reliably detected via immunostaining.

To assess how MEIOC impacts the transcriptome during spermatogenesis, we carried out scRNA-seq-based differential expression analysis in germ cell clusters associated with mitotic spermatogonia (Undiff, A1-4, In/B and B G2M clusters), the mitosis-to-meiosis transition (pL G1, eS and lS clusters) and meiotic prophase I (L and Z clusters), and identified genes whose transcript abundance is increased (log2 fold change wild type/*Meioc* knockout>0.1 and adjusted *P*<0.05) or decreased (log2 fold change wild type/*Meioc* knockout<−0.1 and adjusted *P*<0.05) by MEIOC (Fig. 1C, Table S3). The number of differentially abundant transcripts increased in the In/B and B G2/M clusters, mirroring *Meioc* and *Ythdc2* expression patterns, and continued to increase thereafter. We conclude that MEIOC begins to affect the germline transcriptome before the meiotic G1/S transition.

Based on functional analysis, transcripts upregulated by MEIOC in the In/B through pL lS clusters were enriched for Gene Ontology (GO) annotations related to meiosis (Fig. 1D, Table S4). Several transcripts contributing to this enrichment became more abundant in mitotic spermatogonia and remained more abundant through early meiotic spermatocytes (Fig. 1E). However, in the L and Z clusters, these changes in meiosis-associated transcripts no longer represented an enrichment in meiosis-associated GO annotations (Fig. 1D). Bulk RNA-seq analysis of preleptotene-enriched testes also revealed enrichment of meiosis-associated factors among MEIOC-upregulated transcripts (Fig. S4C,E, Table S11). Therefore, MEIOC activity leads to increased meiotic transcript abundance in late mitotic spermatogonia, earlier than previously appreciated. Misregulation of these transcripts provides a molecular rationale for the delayed progression of *Meioc*-null spermatogenic cells from preleptotene to leptotene to zygotene stages (Abby et al., 2016; Soh et al., 2017).

Given that MEIOC lowers mitotic transcript abundance in fetal oogonia (Soh et al., 2017), we asked whether similar transcriptomic changes were induced by MEIOC in mitotic spermatogonia. MEIOC-downregulated transcripts in the pL lS to Z clusters, but not in developmentally earlier clusters, were enriched for the GO annotation ‘mitotic cell cycle’ (Fig. 1D,E, Table S5). This included mitotic G1/S and G2/M cyclin *Ccna2* (Fig. 1E), the downregulation of which was first evident in B G2/M and pL G1 clusters, before the broader enrichment of mitotic cell cycle-associated transcripts was detected. Consistent with downregulation of *Ccna2* transcript abundance by MEIOC, *Meioc*-null spermatocytes exhibit prolonged CCNA2 protein expression at leptotene and zygotene stages, when wild-type spermatocytes no longer express this protein (Soh et al., 2017). Based on a curated list of genes whose expression is linked to specific cell cycle phases (Hsiao et al., 2020), MEIOC-downregulated genes in pL lS to Z clusters were primarily associated with G2 or M phases (Fig. 1F). We conclude that the downregulation of mitotic cell cycle transcripts by MEIOC occurs primarily after the meiotic G1/S transition. The loss of this regulation likely contributes to the delayed progression of *Meioc*-null spermatogenic cells from preleptotene to leptotene to zygotene stages, and their premature progression into an aberrant metaphase state during meiotic prophase I (Abby et al., 2016; Soh et al., 2017).

In summary, MEIOC elevates the abundance of meiosis-associated transcripts beginning in late transit-amplifying spermatogonia through the mitosis-to-meiosis transition and early meiotic prophase I. Then, late in the mitosis-to-meiosis transition into meiotic prophase I, MEIOC broadly lowers mitotic transcript abundance.

### MEIOC destabilizes transcripts that it targets

**Fig. 2.**
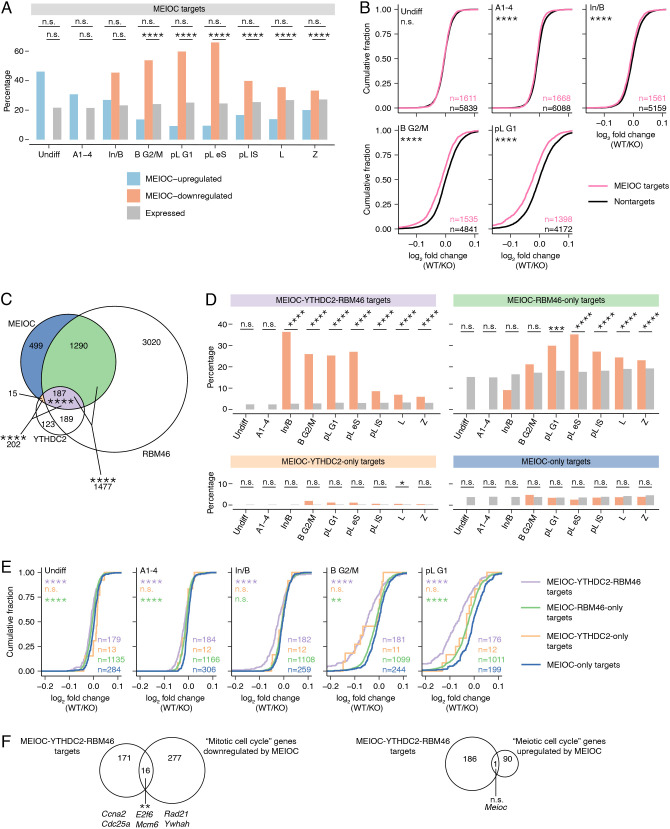
**MEIOC-YTHDC2-RBM46 destabilize their mRNA targets.** (A) Percentage of MEIOC targets within MEIOC-upregulated, MEIOC-downregulated and expressed genes, defined by scRNA-seq. MEIOC targets were identified from re-analysis of a published RIP-seq dataset (Soh et al., 2017). (B) Cumulative fraction for log_2_ fold change (wild type/*Meioc* knockout), defined by scRNA-seq, with genes binned as MEIOC targets or nontargets. (C) Overlap of mRNAs identified by MEIOC RIP-seq, YTHDC2 CLIP-seq and RBM46 eCLIP-seq datasets. MEIOC RIP-seq data were re-analyzed from Soh et al. (2017). YTHDC2 CLIP-seq analysis was published in Saito et al. (2022). RBM46 eCLIP-seq data were re-analyzed from Qian et al. (2022). Asterisks indicate statistical enrichment. (D) Percentage of MEIOC-YTHDC2-RBM46 targets, MEIOC-RBM46-only targets, MEIOC-YTHDC2-only targets and MEIOC-only targets within MEIOC-downregulated and expressed genes. (E) Cumulative fraction for log_2_ fold change (wild type/*Meioc* knockout), defined by scRNA-seq, with genes binned as MEIOC-YTHDC2-RBM46 targets, MEIOC-RBM46-only targets, MEIOC-YTHDC2-only targets and MEIOC-only targets. Asterisks represent the comparison of the color-matched target set to MEIOC-only targets. (F) MEIOC-YTHDC2-RBM46 targets are enriched for MEIOC-downregulated genes annotated with GO term ‘mitotic cell cycle’ but are not enriched for MEIOC-upregulated genes annotated with GO term ‘meiotic cell cycle’. Upregulated and downregulated genes were defined by scRNA-seq (any germ cell cluster). Asterisks represent statistical enrichment. n.s., no significant statistical enrichment or depletion. **Padj.<0.01; ***Padj.<0.0001; ****Padj.<0.0001; n.s., not significant. See Table S1 for details on statistical testing.

In early spermatocytes, MEIOC localizes to the cytoplasm and interacts with the RNA-binding proteins YTHDC2 and RBM46, which recruit other proteins that degrade mRNA (Kretschmer et al., 2018; Li et al., 2022; Qian et al., 2022; Wojtas et al., 2017). Therefore, we hypothesized that MEIOC degrades its mRNA targets. We first defined transcripts that associate with MEIOC by reanalyzing previously published MEIOC RIP-seq data from P15 testes (Soh et al., 2017), identifying 1991 MEIOC-bound mRNA (Table S6). We assessed the molecular impact of the interaction of MEIOC with these transcripts via three approaches. First, we examined the representation of these targets among transcripts exhibiting statistically significant abundance changes that were MEIOC dependent (i.e. MEIOC upregulated or downregulated). We found that MEIOC targets were enriched among transcripts whose abundance decreased, but not those that increased, in response to MEIOC, in the B G2/M through Z clusters (Fig. 2A). Second, we examined all MEIOC targets, irrespective of whether they met a statistical cut-off in the differential expression analysis. MEIOC targets exhibited slightly lower fold changes (wild type/*Meioc* knockout) than nontargets in the A1-4 through Z clusters (Fig. 2B, Fig. S5A). Third, an analysis of estimated changes in transcript stability from bulk RNA-seq data showed that MEIOC targets had reduced transcript stability relative to nontargets (Fig. S6A, Table S7). Therefore, MEIOC lowers the abundance of transcripts that it targets, presumably by promoting their degradation, beginning in mitotic spermatogonia.

Given that MEIOC forms a complex with YTHDC2 and RBM46 (Abby et al., 2016; Li et al., 2022; Qian et al., 2022; Soh et al., 2017), we hypothesized that many MEIOC targets are also bound by YTHDC2 and RBM46. Based on a published YTHDC2 CLIP analysis from testes (Saito et al., 2022) and our re-analysis of RBM46 CLIP data from testes (Qian et al., 2022), MEIOC shared 202 mRNA targets with YTHDC2 and 1477 targets with RBM46, both of which are statistically significant overlaps (Fig. 2C). The three proteins had 187 mRNA targets in common. Only 15 mRNAs were bound by MEIOC and YTHDC2 but not RBM46, and these likely represent technical differences between datasets rather than a biologically meaningful group of transcripts. MEIOC-YTHDC2-RBM46 targets were enriched for GO annotations associated with RNA stability, whereas MEIOC-RBM46-only targets did not exhibit statistically significant enrichment (Table S6). We conclude that MEIOC shares many, but not all, of its mRNA targets with YTHDC2 and RBM46.

As YTHDC2 and RBM46 interact with proteins that degrade mRNA, we hypothesized that MEIOC has a greater impact on transcript stability when acting in partnership with YTHDC2 and RBM46 than when acting alone. To test this, we binned the targets of MEIOC as follows: MEIOC-YTHDC2-RBM46 targets, MEIOC-RBM46-only targets, MEIOC-YTHDC2-only targets and MEIOC-only targets. We compared how these groups of target genes respond to MEIOC in our scRNA-seq data. First, we examined whether each group was over-represented among transcripts whose abundance decreased in response to MEIOC. MEIOC-YTHDC2-RBM46 targets exhibited such enrichment beginning in mitotic spermatogonia (Fig. 2D). By contrast, MEIOC-YTHDC2-only targets were enriched in one meiotic cluster and MEIOC-only targets showed no such enrichment (Fig. 2D). MEIOC-RBM46-only targets were enriched beginning at the mitosis-to-meiosis transition (Fig. 2D), suggesting that MEIOC-RBM46 may impact the transcriptome independently of YTHDC2, but the biological significance of this regulation remains unclear. Second, we examined the effect of YTHDC2 and RBM46 on the stability of MEIOC targets, irrespective of statistical cutoff. Relative to MEIOC-only targets, MEIOC-YTHDC2-RBM46 targets exhibited the largest decrease in transcript abundance in all scRNA-seq germ cell clusters (Fig. 2E, Fig. S5B), as well as in transcript stability in the bulk RNA-seq analysis of preleptotene-enriched testes (Fig. S6B). Taken together, these data demonstrate that the destabilization by MEIOC of its target mRNAs occurs via its interaction with both YTHDC2 and RBM46.

Given that MEIOC-downregulated transcripts are enriched for mitotic cell cycle factors, we hypothesized that MEIOC-YTHDC2-RBM46 directly targets and destabilizes these factors. By comparing MEIOC-downregulated transcripts bearing the GO annotation ‘mitotic cell cycle’ with MEIOC-YTHDC2-RBM46 targets, we discovered a small but statistically significant overlap that included factors that impact cell cycle progression, such as *Ccna2* and *E2f6* (Fig. 2F). Some targets were downregulated by MEIOC in mitotic spermatogonia and early in the mitosis-to-meiosis transition, before mitotic cell cycle enrichment was evident among the MEIOC-downregulated genes (Fig. 1D,E). Therefore, the destabilization of mRNAs by MEIOC before and during the mitosis-to-meiosis transition may impact the ability of spermatogenic cells to establish a meiosis-specific cell cycle program in meiotic prophase I.

We also considered the possibility that some MEIOC-YTHDC2-RBM46 targets are stabilized, rather than destabilized, by the complex. In particular, given that MEIOC-upregulated transcripts are enriched for the ‘meiotic cell cycle’ annotation (Fig. 1D,E), we asked whether these transcripts were MEIOC-YTHDC2-RBM46 targets. MEIOC-upregulated ‘meiotic cell cycle’ transcripts and MEIOC-YTHDC2-RBM46 targets shared one transcript, *Meioc* (Fig. 2F), with this overlap representing neither statistical depletion nor enrichment. We conclude that MEIOC-YTHDC2-RBM46 does not directly regulate the stability of meiosis-associated transcripts. Overall, MEIOC collaborates with YTHDC2 and RBM46 to promote the decay of the transcripts that it targets, beginning in mitotic spermatogonia.

### The destabilization of *E2f6* and *Mga* transcripts by MEIOC elevates expression of E2F6- and MGA-repressed genes

**Fig. 3.**
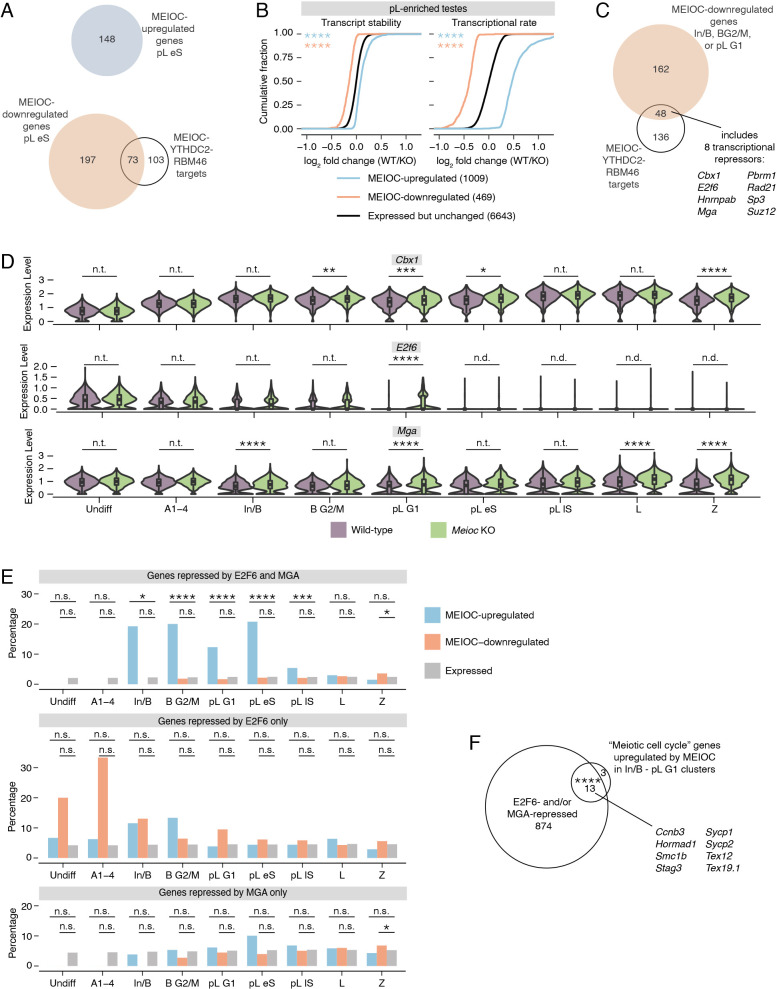
**Inhibition of *E2f6* and *Mga* mRNA by MEIOC-YTHDC2-RBM46 relieves E2F6- and MGA-mediated transcriptional repression.** (A) Venn diagram comparing MEIOC-upregulated and -downregulated genes in pL eS cluster to MEIOC-YTHDC2-RBM46 targets. The majority of genes whose transcript abundance changes in response to MEIOC are not directly targeted by MEIOC-YTHDC2-RBM46. (B) Cumulative distribution of log_2_ fold change (wild type/*Meioc* knockout) for transcript stability (left) and transcriptional rate (right) for MEIOC-upregulated and MEIOC-downregulated genes compared with genes that were expressed but whose expression did not change in response to MEIOC. Transcript stabilities and transcriptional rates were estimated using bulk RNA-seq data from preleptotene-enriched testes. Asterisks represent significant differences between the color-matched gene set to the expressed but not regulated gene set. (C) Identification of MEIOC-YTHDC2-RBM46 targets that are downregulated by MEIOC in In/B, B G2/M and/or pL G1 clusters. Of this set, eight mRNAs encode proteins that have been reported to function as transcriptional repressors. (D) Expression levels for PRC1.6 subunits *Cbx1*, *E2f6* and *Mga* in wild-type versus *Meioc*-null cells in all germ cell clusters identified. *Cbx1*, *E2f6* and *Mga* are downregulated by MEIOC in the In/B, B G2/M and/or pL G1 clusters (*P*adj.<0.05). (E) Percentage of genes repressed by E2F6 and MGA (top), E2F6 only (center) and MGA only (bottom) among MEIOC-upregulated, MEIOC-downregulated and expressed genes. E2F6-repressed genes and MGA-repressed genes were identified via re-analysis of published ChIP-seq and RNA-seq datasets from mouse embryonic stem cells (Dahlet et al., 2021; Stielow et al., 2018; Qin et al., 2021). (F) Overlap between E2F6- and/or MGA-repressed genes and the ‘meiotic cell cycle’ genes upregulated by MEIOC in In/B, B G2/M and pL G1 clusters. Example genes that fall within this overlap are listed. *Padj.<0.05; ***Padj.<0.001; ****Padj.<0.0001; n.s., not significant; n.t., not tested (comparison was excluded from statistical testing because log_2_ fold change>−0.1 and <0.1); n.d., not detected (transcript expressed in fewer than 25% of cells in each population being compared). See Table S1 for details on statistical testing.

Most MEIOC-regulated transcripts, as defined by RNA-seq analysis of wild-type versus *Meioc*-null samples, do not directly interact with MEIOC-YTHDC2-RBM46 (Fig. 3A, Fig. S6C). We examined whether these changes in transcript abundance reflected altered transcriptional rates or altered transcript stabilities. Applying REMBRANDTS to our bulk RNA-seq data, we estimated mRNA abundance from exonic reads, pre-mRNA abundance (i.e. transcriptional rate) from intronic reads, and mRNA stability from the difference between exonic and intronic reads (Alkallas et al., 2017; Gaidatzis et al., 2015). MEIOC-regulated transcripts exhibited large changes in transcriptional rates and smaller changes in transcript stabilities (Fig. 3B). We conclude that MEIOC indirectly impacts transcription in ways that alter the abundance of transcripts that it does not bind.

We set out to identify MEIOC targets that might drive such transcriptional changes. The vast majority of MEIOC-upregulated transcripts are not directly bound by MEIOC (Figs 2A and 3A). We hypothesized that MEIOC-YTHDC2-RBM46 destabilizes a mRNA that encodes a transcriptional repressor; destabilization of this mRNA then derepresses (i.e. upregulates) gene expression. For this analysis, we focused on the In/B to pL G1 clusters, before spermatogenic cells have undergone the MEIOC-dependent transcriptomic shift observed in the scRNA-seq pseudotime analysis (Fig. 1A). Among 48 MEIOC-YTHDC2-RBM46 targets downregulated by MEIOC in the In/B to pL G1 clusters, we identified eight mRNAs that encode transcriptional repressors: *Cbx1*, *E2f6*, *Hnrnpab*, *Mga*, *Pbrm1*, *Rad21*, *Sp3* and *Suz12* (Fig. 3C,D). Strikingly, *Cbx1*, *E2f6* and *Mga* all encode subunits of the noncanonical Polycomb Repressive Complex (PRC) 1.6 (Figs S7A and S9A), representing a statistically significant enrichment for the subunits of the complex (one-tailed hypergeometric test, *P*=3.55E-05). *E2f6* and *Mga* are particularly attractive candidates because they encode sequence-specific DNA-binding subunits of PRC1.6 that are required to repress meiosis-specific genes in somatic and embryonic stem cells (Pohlers et al., 2005; Dahlet et al., 2021; Kehoe et al., 2008; Maeda et al., 2013; Suzuki et al., 2016; Kitamura et al., 2021; Uranishi et al., 2021). By contrast, CBX1 is not strictly required to repress gene expression, as other CBX proteins can compensate for its absence (Ostapcuk et al., 2018).

We hypothesized that the inhibition of *E2f6* and *Mga* by MEIOC-YTHDC2-RBM46 impacts gene expression during spermatogenesis. To test this, we re-analyzed ChIP-seq and RNA-seq datasets from mouse embryonic stem cells (Dahlet et al., 2021; Qin et al., 2021; Stielow et al., 2018) to identify genes directly repressed by E2F6 or MGA (see Materials and Methods; Fig. S8A,B, Table S12). Given that genetic ablation of *Max* (which encodes the binding partner of MGA and a PRC1.6 subunit) induces embryonic stem cells to enter meiosis (Suzuki et al., 2016), these data are relevant to meiotic entry in spermatogenic cells. As E2F6 and MGA cooperate to repress expression of an overlapping set of genes (Dahlet et al., 2021) (Fig. S8C), we classified genes as repressed by both E2F6 and MGA, repressed by E2F6 alone, or repressed by MGA alone. Genes repressed by both E2F6 and MGA, but not those repressed by either factor alone, were over-represented among MEIOC-upregulated transcripts, beginning in the mitotic In/B cluster (Fig. 3E), coinciding with the destabilization of *Mga* mRNA by MEIOC in that cluster (Fig. 3D). This over-representation continued through the mitosis-to-meiosis transition (Fig. 3E), including the pL G1 cluster, when MEIOC destabilizes *E2f6* and *Mga* mRNA (Fig. 3D). Genes repressed by both E2F6 and MGA were not enriched among MEIOC-downregulated transcripts (Fig. 3E). Bulk RNA-seq analysis of preleptotene-enriched testes largely confirmed these results, while also revealing that MEIOC-upregulated genes were enriched for genes repressed by E2F6 or MGA alone (Fig. S9B), likely because this analysis included lowly expressed genes that were not detected in the sparser scRNA-seq dataset. We conclude that the repression of E2F6 and MGA by MEIOC results in enhanced expression of specific genes in mitotic spermatogonia.

We also examined whether the repression of E2F6 and MGA by MEIOC results in diminished expression of other genes in spermatogenic cells. For this analysis, we used the mouse embryonic stem cell datasets to classify genes as activated by both E2F6 and MGA, by E2F6 alone, or by MGA alone. Genes activated by MGA alone were modestly enriched among MEIOC-downregulated genes within a limited set of clusters (pL lS and L; Fig. S8D). By contrast, genes activated by both E2F6 and MGA, or by E2F6 alone were not enriched among MEIOC-upregulated (or downregulated) genes (Fig. S8D). Bulk RNA-seq analysis of preleptotene-enriched testes confirmed these results (Fig. S9B). We conclude that the repression of E2F6 and MGA by MEIOC leads to upregulation of gene expression.

As many MEIOC-upregulated transcripts are meiotic cell cycle factors not bound by MEIOC-YTHDC2-RBM46 (Figs 1D and 2F), we hypothesized that repression of E2F6 and MGA enhances expression of these meiotic genes. To test this, we asked whether E2F6- and MGA-repressed genes are enriched among meiotic cell cycle transcripts upregulated by MEIOC. We again focused this analysis on the In/B to pL G1 clusters, before spermatogenic cells undergo MEIOC-dependent transcriptomic changes. We found that 13 out of 17 MEIOC-upregulated meiotic cell cycle transcripts are targeted for repression by E2F6 and/or MGA (Fig. 3F), and we confirmed these observations in bulk RNA-seq analysis of preleptotene-enriched testes (Fig. S9C). We conclude that the destabilization of *E2f6* and *Mga* mRNAs by MEIOC de-represses meiosis-associated gene expression beginning in mitotic spermatogonia.

### MEIOC indirectly activates the transcriptional regulator STRA8-MEIOSIN to enhance the competence of spermatogenic cells to initiate meiosis

**Fig. 4.**
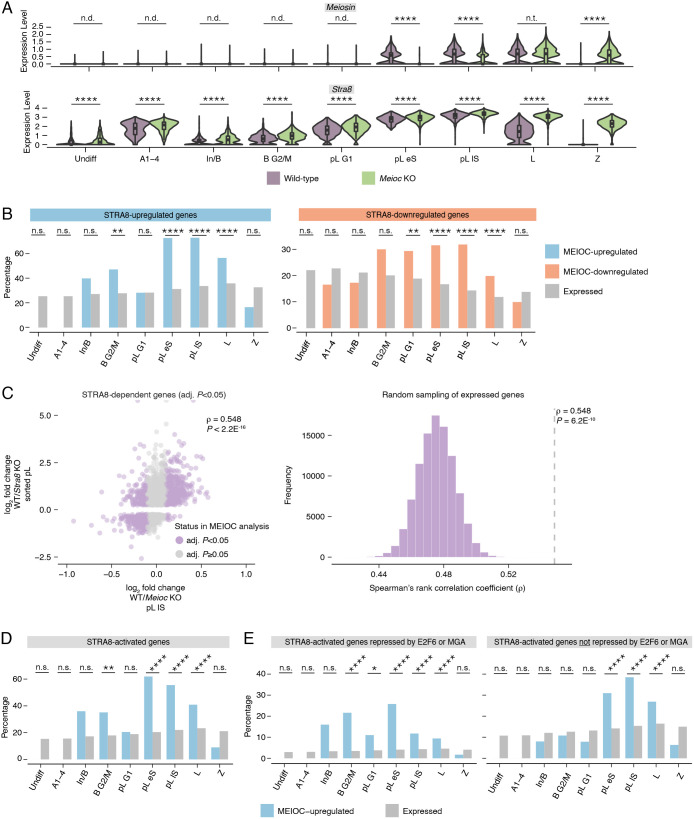
**Derepression of *Meiosin* gene expression by MEIOC enhances activation of the STRA8-MEIOSIN transcriptional program.** (A) Expression levels of *Meiosin* and *Stra8* in wild type and *Meioc* knockout in all germ cell clusters. (B) Percentage of STRA8-upregulated and -downregulated genes in MEIOC-upregulated, -downregulated and expressed genes from scRNA-seq analysis. STRA8-upregulated and -downregulated genes were identified via re-analysis of bulk RNA-seq data from wild-type and *Stra8* knockout sorted preleptotene spermatocytes from Kojima et al. (2019). (C) Left: correlation between MEIOC scRNA-seq analysis of the pL lS cluster and STRA8 bulk RNA-seq analysis of sorted preleptotene spermatocytes. Analysis was limited to genes that were statistically dependent on STRA8 (*P*adj.<0.05). *P*-value represents the probability that Spearman rho does not equal 0. Right: distribution of correlations for gene sets obtained by random sampling of genes expressed in the scRNA-seq pL lS cluster and bulk RNA-seq sorted preleptotene spermatocytes. *P*-value represents that probability of obtaining an equal or larger correlation by random sampling. (D) Percentage of STRA8-activated genes in MEIOC-upregulated and expressed genes from scRNA-seq analysis. STRA8-activated genes were identified as those genes with STRA8-bound promoters (as identified by Kojima et al., 2019 via STRA8-FLAG ChIP-seq in preleptotene-enriched testes) and upregulated by STRA8 (as identified by re-analysis of bulk RNA-seq data from wild-type and *Stra8* knockout sorted preleptotene spermatocytes from Kojima et al., 2019). (E) Percentage of STRA8-activated genes repressed by E2F6 or MGA (left), as well as STRA8-activated genes not repressed by E2F6 or MGA (right), in MEIOC-upregulated and expressed genes. *Padj.<0.05; **Padj.<0.01; ****Padj.<0.0001; n.s., not significant; n.t., not tested (comparison was excluded from statistical testing because log_2_ fold change> −0.1 and <0.1); n.d., not detected (transcript expressed in fewer than 25% of cells in each population being compared). See Table S1 for details on statistical testing.

In mouse embryonic stem cells, E2F6 and MGA directly repress *Meiosin* (previously known as *Gm4969* and *Bhmg1*) (Uranishi et al., 2021) (Fig. S8A,B). Consistent with the destabilization of *E2f6* and *Mga* mRNAs by MEIOC, we found that MEIOC enhances *Meiosin* expression at both the transcript and protein levels during the mitosis-to-meiosis transition (Fig. 4A, Figs S4C, S10A, Table S13). By meiotic prophase I, *Meioc*-null germ cells exhibited delayed upregulation of *Meiosin* expression (Fig. 4A). We confirmed that *Meiosin* expression is also increased by YTHDC2 and RBM46, based on our examination of published RNA-seq data from postnatal testes (Jain et al., 2018; Peart et al., 2022). MEIOC protein does not bind *Meiosin* mRNA (Table S6). This suggests that the regulation of *E2f6* and *Mga* by MEIOC-YTHDC2-RBM46 may be impacting *Meiosin* gene expression.

We examined whether MEIOC affects other known regulators of *Meiosin* gene expression. As retinoic acid transcriptionally activates *Meiosin* (Ishiguro et al., 2020), we considered whether MEIOC enhances retinoic acid-mediated transcription, but we found no evidence for this model. MEIOC did not increase the transcript abundance of any retinoic acid receptors (RARs) or retinoid X receptors (RXRs), which mediate transcriptional activation by retinoic acid (reviewed by Endo et al., 2019) (Fig. S10B,C). In addition, MEIOC did not upregulate additional retinoic acid-activated genes, such as *Stra8*, which encodes the binding partner of MEIOSIN, and *Rec8*, a meiotic cohesin (Koubova et al., 2014; Soh et al., 2015; Zhang et al., 2021) (Fig. 4A, Fig. S10C,D). We observed that MEIOC downregulated *Stra8* abundance overall in the scRNA-seq data (Fig. 4A), but as *Stra8* mRNA is not directly bound by MEIOC (Table S6), the molecular basis for this regulation remains uncharacterized. These observations indicate that MEIOC does not activate *Meiosin* gene expression by increasing retinoic acid-mediated transcription.

We also considered whether *Dmrt1*, encoding a protein that presumably represses *Meiosin* gene expression (Ishiguro et al., 2020), was regulated by MEIOC-YTHDC2-RBM46; again, we found no evidence for this alternative model. *Dmrt1* transcript was not a target of MEIOC, YTHDC2 or RBM46 (Table S6), nor was it differentially expressed in response to MEIOC in the pL G1 or pL eS clusters of the scRNA-seq analysis or in the preleptotene-enriched testes of the bulk RNA-seq analysis (Fig. S11A,B). DMRT1 protein expression was similar in both wild-type and *Meioc*-null testes, with DMRT1 expressed in mitotic spermatogonia but absent from preleptotene spermatocytes (Fig. S11C). In addition, MEIOC did not affect the expression of DMRT1-regulated genes *Tbx1* and *Crabp2* (Matson et al., 2010) (Fig. S11D,E). DMRT1 also directly inhibits *Stra8* expression (Matson et al., 2010), but as noted above, *Stra8* was not activated by MEIOC (Fig. 4A). Therefore, changes in DMRT1 do not account for the upregulation of *Meiosin* gene expression by MEIOC. Taking these observations together, we conclude that the inhibition of *E2f6* and *Mga* by MEIOC derepresses *Meiosin* gene expression during the mitosis-to-meiosis transition.

STRA8-MEIOSIN functions as an obligate heterodimer that transcriptionally activates gene expression during the mitosis-to-meiosis transition (Ishiguro et al., 2020; Kojima et al., 2019). *Stra8* is highly expressed at both the transcript and protein levels in *Meioc*-null spermatogenic cells at the mitosis-to-meiosis transition (Fig. 4A, Figs S4C and S10A), as previously reported (Abby et al., 2016; Soh et al., 2017). We hypothesized that the upregulation of *Meiosin* gene expression by MEIOC leads to STRA8-MEIOSIN-mediated transcriptional changes. We tested three predictions of this hypothesis.

First, we tested whether, during the mitosis-to-meiosis transition, genes dependent on MEIOC also depend on STRA8. We defined STRA8-dependent (i.e. STRA8-upregulated or -downregulated) genes using bulk RNA-seq data from wild-type and *Stra8*-null preleptotene spermatocytes isolated via synchronization and sorting (Kojima et al., 2019). STRA8-upregulated genes were enriched among MEIOC-upregulated genes during the mitosis-to-meiosis transition (pL eS and lS clusters) and in meiotic prophase I (L cluster; Fig. 4B). Similarly, STRA8-downregulated transcripts were enriched among MEIOC-downregulated transcripts at these same stages (Fig. 4B). Bulk RNA-seq analysis of preleptotene-enriched testes produced similar results (Fig. S12A).

Second, focusing exclusively on STRA8-dependent genes, we looked for correlated changes in transcript abundance in the MEIOC scRNA-seq and STRA8 bulk RNA-seq datasets. STRA8-dependent genes exhibited significantly correlated effects in the MEIOC and STRA8 datasets that peaked at the mitosis-to-meiosis transition (pL eS and lS clusters; Fig. 4C, Figs S13A, S14A). STRA8-dependent genes similarly exhibited robustly correlated effects in the MEIOC bulk RNA-seq dataset from preleptotene-enriched testes and the STRA8 dataset (Fig. S12B). We conclude that MEIOC activity leads to STRA8-MEIOSIN-mediated changes in transcript abundance.

Third, we tested whether MEIOC-dependent genes are directly activated by STRA8-MEIOSIN. We identified STRA8-activated genes as those genes (1) whose expression increased in response to STRA8 and (2) whose promoters were bound by STRA8 (as defined by ChIP-seq from testes enriched for preleptotene spermatocytes; Kojima et al., 2019). We found that MEIOC-upregulated genes were enriched for STRA8-activated genes during the mitosis-to-meiosis transition (pL eS and lS clusters; Fig. 4D). However, *de novo* motif analysis failed to identify enrichment of any motif among these MEIOC-upregulated genes, likely due to the sparsity of scRNA-seq data. The enrichment of STRA8-activated genes among MEIOC-upregulated genes was also visible in the bulk RNA-seq analysis of preleptotene-enriched testes (Fig. S12C). Furthermore, the top motif identified by *de novo* motif analysis of MEIOC-upregulated genes from the bulk RNA-seq analysis matched the STRA8-MEIOSIN binding motif (Fig. S12D). The second top motif matched the binding site shared by E2F6 and other E2F proteins (Fig. S12D), as previously reported among STRA8-activated genes (Kojima et al., 2019). In total, ∼60-70% of MEIOC-upregulated genes are directly activated by STRA8-MEIOSIN.

Together, these analyses demonstrate that MEIOC indirectly activates the STRA8-MEIOSIN transcriptional regulator, thereby facilitating a massive shift in the transcriptome at the meiotic G1/S transition (Fig. 1A). With this new molecular insight, we revisited our interpretation of the *Meioc*-null phenotype. Based on previous histological analyses and DNA labeling experiments, *Meioc*-null spermatogenic cells successfully enter the preleptotene stage, express STRA8 protein and exhibit DNA synthesis. However, they then accumulate at the preleptotene stage and are delayed in entering meiotic prophase I (Abby et al., 2016; Soh et al., 2017). Previously, we interpreted these data as *Meioc*-null spermatogenic cells successfully undergoing the meiotic G1/S phase transition but thereafter exhibiting disrupted progression into meiotic prophase I. Given our present findings that *Meioc*-null germ cells are delayed in upregulating *Meiosin* expression and exhibit a disrupted meiotic G1/S phase transition, we now interpret the *Meioc*-null phenotype as defective meiotic initiation. We conclude that MEIOC enhances the competence of spermatogenic cells to initiate meiosis by activating the STRA8-MEIOSIN transcriptional regulator.

### MEIOC derepresses meiotic gene expression before activating STRA8-MEIOSIN

We observed that many STRA8-activated genes are repressed by E2F6 and/or MGA (Fig. S12E). Consistent with this observation, STRA8-activated genes are enriched for the binding motif of E2F6 and other E2F proteins (Fig. 12D; Kojima et al., 2019). Accordingly, we divided STRA8-activated genes into two groups, based on whether or not they were directly repressed by E2F6 and/or MGA in embryonic stem cells. As MEIOC represses E2F6 and MGA in mitotic spermatogonia and activates STRA8-MEIOSIN during the mitosis-to-meiosis transition, we predicted that genes experimentally defined as E2F6- and/or MGA-repressed and STRA8-activated would be upregulated by MEIOC, starting in mitotic spermatogonia, coincident with the destabilization of *E2f6* and *Mga* mRNAs by MEIOC. Conversely, STRA8-activated genes not repressed by E2F6 or MGA should be upregulated by MEIOC later in the mitosis-to-meiosis transition, coincident with MEIOC-mediated upregulation of *Meiosin* gene expression. Consistent with these predictions, STRA8-activated, E2F6- and/or MGA-repressed genes were enriched among MEIOC-upregulated genes in the mitotic B G2/M cluster and during the mitosis-to-meiosis transition (pL G1, eS and lS clusters; Fig. 4E). Also as predicted, STRA8-activated genes not repressed by E2F6 or MGA exhibited enrichment in the pL eS and lS clusters (Fig. 4E). Both genesets were also enriched among MEIOC-upregulated genes in the bulk RNA-seq analysis (Fig. S12F). We conclude that, during late transit amplification, MEIOC derepresses a set of meiotic genes targeted by E2F6 and MGA; then, during the mitosis-to-meiosis transition, MEIOC elevates expression of these and other meiotic genes by activating STRA8-MEIOSIN.

**Fig. 5.**
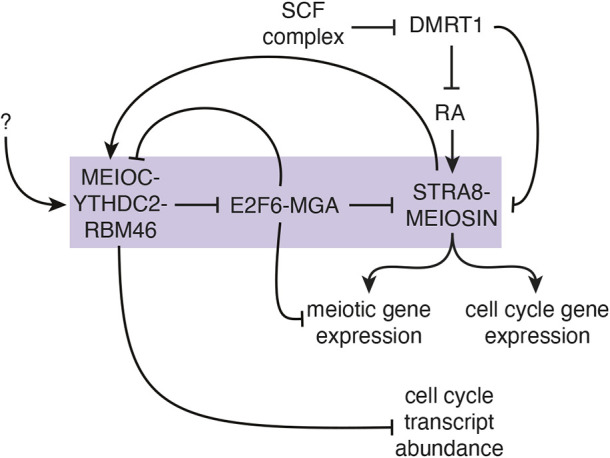
**Model for MEIOC-YTHDC2-RBM46 enhancing the competence of spermatogenic cells to transition from mitosis to meiosis in response to retinoic acid.** MEIOC-YTHDC2-RBM46 destabilize *E2f6* and *Mga* mRNA, and thereby inhibit E2F6- and MGA-mediated repression of transcription at genes involved in meiosis, including *Meiosin* and *Meioc*. In parallel, the SCF complex degrades DMRT1 and consequently inhibits the repression of retinoic acid (RA)-dependent transcription by DMRT1 as well as *Stra8* and *Meiosin* gene expression. Retinoic acid activates *Stra8* and *Meiosi*n gene expression. This activates the STRA8-MEIOSIN transcription factor, which drives the transcription of cell cycle genes as well as meiotic genes, many of which were previously repressed by E2F6 and MGA. The repression of cell cycle transcripts by MEIOC also contributes to the establishment of a meiosis-specific cell cycle program in meiotic prophase I. The question mark indicates undefined molecular regulation that activates MEIOC-YTHDC2-RBM46 before retinoic acid activates STRA8-MEIOSIN transcriptional activity and the transition from mitosis to meiosis. Purple box highlights the previously unreported regulation identified in this study that facilitates competence for the mitosis-to-meiosis transition.

In total, the destabilization of *E2f6* and *Mga* mRNAs by MEIOC-YTHDC2-RBM46 in late mitotic spermatogenic cells derepresses E2F6-MGA-targeted genes, including *Meiosin* and other meiosis-associated genes. In turn, these steps drive activation of the STRA8-MEIOSIN transcriptional regulator by retinoic acid during the mitosis-to-meiosis transition (Fig. 5). As STRA8-MEIOSIN is the molecular vehicle through which retinoic acid induces the meiotic G1/S transition, the regulation of the STRA8-MEIOSIN complex by MEIOC enhances competence of spermatogenic cells to initiate meiosis.

## DISCUSSION

Here, we demonstrate that *Meioc*-null spermatogenic cells developmentally diverge from their wild-type counterparts during the meiotic G1/S transition, earlier than previously appreciated. MEIOC is required to derepress expression of meiotic genes, including the transcriptional regulator *Meiosin*. In turn, MEIOC elevates expression of genes targeted by STRA8-MEIOSIN, which drives meiotic initiation. Therefore, MEIOC enhances the competence of spermatogenic cells to activate the meiotic transcriptional regulator and initiate meiosis in response to retinoic acid.

We find that MEIOC-YTHDC2-RBM46 destabilizes its mRNA targets, as suggested previously based on analyses of transcript abundance (Hsu et al., 2017; Qian et al., 2022; Saito et al., 2022; Soh et al., 2017). Here we distinguished changes in transcription versus transcript stability using two approaches. First, scRNA-seq allowed us to identify and analyze spermatogenic cells impacted by MEIOC before the onset of major transcriptional changes. Second, we employed a specialized pipeline to distinguish between transcriptional rate and RNA stability in bulk RNA-seq data of preleptotene-enriched testes (Alkallas et al., 2017). These complementary methods confirmed that MEIOC-YTHDC2-RBM46 reduces the stability of its target transcripts. Transcript stability may be the primary mechanism by which MEIOC-YTHDC2-RBM46 regulates its targets, as a recent study using ribosome profiling found that YTHDC2 does not affect translation in postnatal testes (Saito et al., 2022).

MEIOC-YTHDC2-RBM46 elevates the abundance of meiosis-associated transcripts without binding them. Here, we have demonstrated that these transcript abundance changes are driven by changes in transcription (Fig. 3B). Further, we provide a two-step mechanism for these indirect effects on transcript abundance. First, MEIOC-YTHDC2-RBM46 binds to and destabilizes *E2f6* and *Mga* mRNAs, which encode transcriptional repressors whose genomic targets are enriched for meiosis-associated genes (Dahlet et al., 2021; Kehoe et al., 2008; Kitamura et al., 2021; Maeda et al., 2013; Pohlers et al., 2005; Suzuki et al., 2016; Uranishi et al., 2021). This regulation impacts gene expression and potentially differentiation, beginning in late mitotic spermatogonia, when MEIOC begins to destabilize *Mga* (Fig. 3D). Second, this MEIOC-YTHDC2-RBM46-mediated inhibition of *E2f6* and *Mga* derepresses *Meiosin* gene expression and activates STRA8-MEIOSIN. This transcriptional regulator then activates meiotic gene expression and the meiotic G1/S transition. Thus, the inhibition of a transcriptional repressor by MEIOC-YTHDC2-RBM46 confers developmental competence to initiate meiosis.

MEIOC is also required in meiotic oocytes for progression through early meiotic prophase I (Abby et al., 2016; Soh et al., 2017), and it remains an unanswered question whether MEIOC supports activation of STRA8-MEIOSIN during meiotic initiation in premeiotic oogonia. There are some molecular differences in meiotic initiation between oogonia and spermatogenic cells. At stages when wild-type germ cells are undergoing meiotic DNA replication, *Stra8*-null oogonia fail to initiate any DNA replication, whereas *Stra8*-null spermatogenic cells initiate a DNA replication that is non-meiotic in nature, as meiotic cohesin REC8 is not loaded onto the chromosomes (Anderson et al., 2008; Baltus et al., 2006; Dokshin et al., 2013). STRA8 has the additional function of sequestering RB1, a key regulator of the G1/S transition, to promote a timely meiotic G1/S transition in oogonia, but this activity does not appear to impact spermatogenesis (Shimada et al., 2023).

In mitotic oogonia, loss of PRC1 activity via genetic ablation of *Ring1* and *Rnf2* causes premature meiosis due to precocious expression of *Stra8* and other meiosis-associated genes (Yokobayashi et al., 2013). In addition, genetic ablation of *Max*, which encodes a subunit of PRC1.6, induces a similar phenotype with precocious expression of *Stra8*, *Meiosin* and meiosis-associated genes (Suzuki et al., 2024)*.* During spermatogenesis, PRC1.6 may also regulate gene expression and meiotic initiation, but the germline roles for PRC1.6, E2F6 or MGA remain uncharacterized. Although *E2f6*-null mice are fertile, spermatogenesis was reportedly disrupted, without detailed characterization (Storre et al., 2002). Genetic ablation of *Mga* causes embryonic lethality, precluding analysis of spermatogenesis (Burn et al., 2018; Washkowitz et al., 2015). Further studies will be required to characterize the roles of E2F6 and MGA in spermatogenesis, including whether loss of E2F6 and MGA rescues the developmental competence of *Meioc*-null spermatogenic cells. Other transcriptional repressors targeted by MEIOC-YTHDC2-RBM46 may also control *Meiosin* gene expression.

As some *Meioc*-null spermatogenic cells upregulate *Meiosin* gene expression and enter meiotic prophase I on a delayed timeline, MEIOC is not strictly required for competence to initiate meiosis. Perhaps residual activity of YTHDC2 and RBM46 in the absence of MEIOC can support meiotic initiation in some cells. Consistent with this possibility, YTHDC2 protein is still expressed in *Meioc*-null spermatogenic cells (Abby et al., 2016; Soh et al., 2017), but whether loss of MEIOC affects RBM46 protein expression remains unclear. Alternatively, acquisition of competence mediated by the SCF complex may enable meiotic initiation on this delayed timeline. Regardless, we have found that the *Meioc*-null, *Ythdc2*-null or *Rbm46*-null phenotype of delayed entry into meiotic prophase I is the result of reduced STRA8-MEIOSIN transcriptional activation and is less severe than the arrest at the G1/S transition, before meiotic prophase I, exhibited by *Stra8*-null or *Meiosin*-null spermatogenic cells on an inbred C57BL/6 background. Intriguingly, *Stra8*-null spermatogenic cells on a mixed genetic background exhibit the less severe phenotype seen with *Meioc*-null, *Ythdc2*-null or *Rbm46*-null spermatogenic cells. Our analyses of the *Meioc*-null phenotype suggest that, on a mixed genetic background, *Stra8*-null spermatogenic cells transcriptionally activate some meiotic gene expression, perhaps due to MEIOSIN acting as a homodimer, but further studies will be required to test this possibility.

Our model of mammalian meiotic initiation exhibits parallels to the molecular network that governs meiotic initiation in budding yeast. In yeast, inhibition of a transcriptional repressor (Rme1p) via mRNA destabilization activates expression of the key transcription factor (Ime1p) that governs meiotic entry (Blumental-Perry et al., 2002; Bushkin et al., 2019; Covitz et al., 1991; Kahana et al., 2010; Kassir et al., 1988; Mitchell and Herskowitz, 1986; Smith et al., 1990). We conclude that in both unicellular eukaryotes and multicellular organisms, destabilization of a transcriptional repressor at the transcript level controls activation of the meiotic transcriptional program.

We have placed transcriptional activation by STRA8-MEIOSIN and post-transcriptional repression of mRNA by MEIOC-YTHDC2-RBM46 in a positive-feedback loop that facilitates the mitosis-to-meiosis transition (Fig. 5). This model generates a new question: how is MEIOC-YTHDC2-RBM46 activated before retinoic acid activates STRA8-MEIOSIN, particularly when E2F6 and MGA repress *Meioc* gene expression (Fig. 5)? One possibility is illustrated by the *Drosophila* MEIOC homolog Bam, which (translationally) represses its mRNA targets in late mitotic spermatogonia to facilitate the transition from mitosis to meiosis (Insco et al., 2009, 2012). This transition requires that Bam protein accumulates to a critical threshold level (Insco et al., 2009). Whether mammalian MEIOC protein levels must also clear a critical threshold to activate the MEIOC-YTHDC2-RBM46 complex requires further investigation.

In conclusion, by destabilizing its mRNA targets, MEIOC-YTHDC2-RBM46 inhibits transcriptional repressors E2F6 and MGA, thereby allowing spermatogenic cells to activate *Meiosin* expression in response to retinoic acid. In turn, this activates the key meiotic transcriptional regulator STRA8-MEIOSIN, which amplifies expression of meiosis- and cell cycle-associated genes, and drives the meiotic G1/S transition. In total, the post-transcriptional activity of MEIOC-YTHDC2-RBM46 enhances the activity of the meiotic transcriptional regulator. This regulatory pathway, acting in parallel with SCF complex-mediated degradation of DMRT1, enhances the competence of spermatogenic cells to initiate meiosis in response to retinoic acid.

## MATERIALS AND METHODS

### Animals

All experiments involving mice were performed in accordance with the guidelines of the Massachusetts Institute of Technology (MIT) Division of Comparative Medicine and Cincinnati Children's Hospital Medical Center (CCHMC) Division of Veterinary Services, which are overseen by their respective Institutional Animal Care and Use Committees (IACUC). The animal care programs at MIT/Whitehead Institute and CCHMC are accredited by the Association for Assessment and Accreditation of Laboratory Animal Care, International (AAALAC) and meet or exceed the standards of AAALAC as detailed in the Guide for the Care and Use of Laboratory Animals. This research was approved by the MIT IACUC (0617-059-20) and CCHMC IACUC (2022-0061).

Mice carrying the *Meioc*-null allele *Meioc^tm1.1Dpc^* (Soh et al., 2017) were backcrossed to C57BL/6N (B6N) from Taconic Biosciences for at least 10 generations. Mice used for scRNA-seq experiments were also heterozygous for *Hspa2^tm1Dix^* (RRID: IMSR_JAX:013584; Dix et al., 1996) and homozygous for *Gt(ROSA)26Sor^tm9(CAG-tdTomato)Hze^* (*ROSA26^tdTomato^*; RRID: IMSR_JAX:007909; Madisen et al., 2010) with the floxed stop codon intact; both of these genotypes exhibit normal spermatogenesis.

### 10x Genomics single-cell RNA-seq

Single-cell sequencing libraries were prepared and sequenced in two batches, with each batch containing one wild-type and one *Meioc*-null pup at P15. One P15 testis per pup was enzymatically dissociated into single cells (see supplementary Materials and Methods) and resuspended in 0.05% bovine serum albumin (BSA) in phosphate-buffered saline (PBS) for a target concentration of 1000 cells per microliter. Cell suspensions were loaded onto the Chromium Controller, aiming for recovery of 10,000 cells per sample. Libraries were generated using the Chromium Next GEM Single Cell 3′ v3.1 (10x Genomics), according to manufacturer's instructions, and sequenced as 150 bp paired-end reads on an Illumina NovaSeq 6000 system with an S4 flow cell.

### Analysis of 10x Genomics scRNA-seq data

Alignment, filtering, barcode counting and UMI counting were carried out using the *count* function in Cell Ranger v.4.0.0 with default settings using Cell Ranger's mm10-2020-A reference package (i.e. the GRCm38/mm10 mouse genome assembly with GENCODE vM23/Ensembl 98 annotation). Using Seurat v.3.2.3 (Stuart et al., 2019), cells were filtered for less than 10% mitochondrial reads, more than 1000 detected features and a doublet score (generated by the *bcds* in scds v.1.2.0) of less than 0.4. Using protein-coding genes, UMI counts from both wild-type and *Meioc*-null samples were integrated and clusters identified using the first 30 dimensions.

The wild-type samples were used to assign cell types to clusters. Five somatic cell types were identified based on cell type-enriched gene expression: fetal Leydig cells (*Dlk1* and *Cyp11a1*) (Kaftanovskaya et al., 2015; Ye et al., 2017), peritubular myoid cells (*Acta2* and *Myh11*) (Chen et al., 2016; Cool et al., 2008), vascular endothelium (*Tm4sf1*) (Shih et al., 2009), testicular macrophages (*Cd14*, *Adgre1* and *Itgam*) (Bhushan et al., 2020; Landmann et al., 2000) and Sertoli cells (*Sox9* and *Cldn11*) (Mazaud-Guittot et al., 2010; Sekido et al., 2004). Germ cells were identified via *Ddx4* and *Dazl*. Then, using additional cell type-enriched gene expression along with UMAP-based cluster relationships, subclusters of spermatogenic cells were assigned to the following cell types: spermatogonial stem cells (*Id4*, *Gfra1* and *Etv5*) (He et al., 2007; Helsel et al., 2017; Sun et al., 2015; Wu et al., 2011), undifferentiated spermatogonia (*Zbtb16*) (Hobbs et al., 2012), type A spermatogonia (*Kit*, *Stra8* and *Ccnd2*) (Beumer et al., 2000; Endo et al., 2015; Schrans-Stassen et al., 1999; Yoshinaga et al., 1991), intermediate/type B spermatogonia (*Kit*) (Schrans-Stassen et al., 1999; Yoshinaga et al., 1991), preleptotene spermatocytes (*Stra8*), leptotene and zygotene spermatocytes (*Meiob*) (Chen et al., 2018; Souquet et al., 2013) and pachytene spermatocytes (*Piwil1*) (Chen et al., 2018; Deng and Lin, 2002).

Spermatogenic clusters were further refined based on cell cycle phase using the *CellCycleScoring* function in Seurat to identify type B spermatogonia in G2/M phase, as well as preleptotene spermatocytes in G1, early S and late S phases (Fig. S1C,D). Clusters were merged as needed. Cell cycle designations of the preleptotene clusters were confirmed via gene expression patterns in wild-type cells independently of the gene:cell cycle phase pairings used by Seurat's *CellCycleScoring* function (Fig. S3). These final cell type designations were then applied to the *Meioc*-null samples. The mutant-only (Mut) cluster was primarily composed of *Meioc*-null spermatocyte cells. Four wild-type germ cells assigned to the Mut cluster (Table S8) were considered to be an scRNA-seq artifact and were thus excluded from subsequent analysis.

In addition to the somatic and germ cell clusters shown in Fig. S1A,B, one additional somatic cell cluster and one additional germ cell cluster were identified but could not be assigned a cell type using marker expression. This unassigned germ cell cluster is likely a technical artifact as it exhibits a median number of features lower than that of all other assigned germ cell subpopulations (Fig. S2A). The unassigned somatic and germ cell clusters, along with all other somatic clusters, were excluded from subsequent analyses. See supplementary Materials and Methods for additional details on cluster-specific enrichment or depletion in wild-type cells and Gene Set Enrichment Analysis (GSEA).

Pseudotime trajectories were built in Monocle 3 v1.0.0 (Qiu et al., 2017a,b; Trapnell et al., 2014) using the following procedure: the Seurat object containing germ cell clusters only was imported as a Monocle object; data were normalized and pre-processed [function *preprocess_cds()*; options: num_dim=100]; batch effects were removed [function *align_cds()*; options: alignment_group=’batch’]; dimensionality reduction was carried out via UMAP [function *reduce_dimension()*]; cells were clustered [function *cluster_cells()*]; a principal graph was learned from the reduced dimension space [function *learn_graph()*]; and cells were ordered by selecting the Undiff cluster as the starting branch of the trajectory [function *order_cells()*]. Cluster cell-type assignments made in Seurat were maintained in the pseudotime trajectories.

For differential expression analysis between wild-type and *Meioc*-null germ cells, log_2_ fold change was defined as wild-type over *Meioc*-null germ cells, such that the value reflects the activity of MEIOC in the unperturbed wild-type state. Differential expression analysis of scRNA-seq data was carried out on each germ cell cluster on genes with a minimum absolute log_2_ fold change of 0.1 and that were detected in at least 25% of either population using Seurat's *FindMarkers* function (options: logfc.threshold=0.1, min.pct=0.25); *P* values were adjusted for multiple hypothesis testing of tested genes across all nine germ cell clusters via the Bonferroni correction using the *P.adjust* function in R. Additional expressed genes (i.e. genes detected in at least 25% of cells in wild-type or *Meioc*-null cells per cluster) that failed to meet the log_2_ fold change threshold for differential expression testing were identified via the *FindMarkers* function (options: logfc.threshold=0, min.pct=0.25) and their *P* values were marked as ‘nd’ for differential expression testing ‘not done’ (see supplementary Materials and Methods for additional details on Gene Ontology analysis, cell cycle analysis of differentially expressed genes and *de novo* motif analysis). Dot plots were generated using Seurat's *DotPlot* function with parameter scale=FALSE to maintain average expression from wild-type and *Meioc*-null samples on the same scale. Code for scRNA-seq analysis is available on GitHub (https://github.com/Mikedis-Lab/2024_Meioc_spermatogenesis/).

### Synchronization of spermatogenesis

Spermatogenesis was synchronized using a protocol originally developed by Hogarth et al. (2013) and modified by Romer et al. (2018) (see supplementary Materials and Methods for details).

### Bulk RNA-seq analysis of preleptotene-enriched testes

Total RNA was extracted from four wild-type samples and three *Meioc*-null samples, using 1.5 synchronized testes from a single pup per sample. TRIzol Reagent (Thermo Fisher Scientific) was added to freshly thawed whole testes, and ERCC RNA ExFold RNA Spike-In Mix 1 and 2 (Thermo Fisher Scientific) were added to wild-type and *Meioc*-null samples, respectively, at a concentration of 1 µl of a 1:100 dilution of spike-in mix per 1 mg of testis tissue. (Spike-in mixes were ultimately not used for data analysis.) Total RNA was then isolated with chloroform following the manufacturer's protocol, precipitated using isopropanol and resuspended in RNase-free water. RNA-seq libraries were prepared with the TruSeq Stranded Total RNA kit with the Ribo-Zero Gold rRNA Removal kit. The barcoded libraries were pooled and sequenced with 50 bp single-end reads on an Illumina HiSeq 2500 machine.

Reads were quality trimmed using cutadapt v1.8 (options: -q 30 --minimum-length 20 -b AGATCGGAAGAGC). Expression levels of all transcripts in the mouse Gencode Basic vM15 gene annotation were estimated using kallisto v0.44.0 (Bray et al., 2016) with sequence-bias correction (--bias) and strand-specific pseudoalignment (--rf-stranded). Quantified transcripts were filtered for protein-coding genes, transcript-level estimated counts and transcripts per million (TPM) values were summed to the gene level, and TPMs were renormalized to transcript-per-million units.

To identify the MEIOC-dependent differential expression program, read counts from kallisto were rounded to the nearest integer and then supplied to DESeq2 v1.26.0 (Love et al., 2014). Genes were filtered for a minimum TPM of 1 in at least three out of seven samples. Differential expression was defined using a cutoff of adjusted *P* value <0.05. See supplementary Materials and Methods for additional details on cell cycle analysis, Gene Ontology analysis and *de novo* motif analysis.

For analysis of transcript stability and transcriptional rates, reads were mapped to the mouse genome (mm10) with the GENCODE Basic vM15 gene annotation via STAR v2.7.1a (Dobin et al., 2013) (options: --outFilterMultimapNmax 1 --alignEndsType Extend5pOfRead1 --outFilterMismatchNmax 2 --outSAMattributes None). All other parameters were set to default. Counts were quantified by htseq v0.11.0 (options: -m union --stranded=reverse) at the gene level (-type gene) and exon level (-type exon), and intron levels were calculated as gene-level counts minus exon-level counts. Changes in transcript stability, transcriptional rate and abundance were calculated for each gene for each sample using REMBRANDTS (Alkallas et al., 2017) with a stringency of 0.80 and linear bias mode.

The cumulative distribution of the log_2_ fold change (wild type/*Meioc* knockout) in transcript stability or transcriptional rate in MEIOC-upregulated or -downregulated transcripts was compared with expressed but unchanged transcripts via a two-tailed Wilcoxon rank sum test with Bonferroni correction for multiple hypothesis testing using the *wilcox.test* and *P.adjust* functions in R. MEIOC-upregulated genes were defined as log_2_ fold change wild type/*Meioc* knockout>0 and *P*<0.05 and MEIOC-downregulated genes were defined as log_2_ fold change wild type/*Meioc* knockout<0 and *P*<0.05 based on the DESeq2 analysis of the bulk RNA-seq data.

### Re-analysis of STRA8 RNA-seq dataset

RNA-seq data from *Stra8*-null and *Stra8*-heterozygote (i.e. phenotypically wild-type) preleptotene spermatocytes (NCBI GEO GSE115928; Kojima et al., 2019) were re-analyzed. Genes with STRA8-bound promoters identified by ChIP-seq in testes enriched for preleptotene spermatocytes were extracted from Kojima et al. (2019) (supplementary file 2: https://doi.org/10.7554/eLife.43738.028) (see supplementary Materials and Methods for details).

### Re-analysis of MEIOC RIP-seq dataset, comparison to YTHDC2 and RBM46 CLIP datasets, and comparison to MEIOC scRNA-seq and bulk RNA-seq datasets

MEIOC RIP-seq from P15 testes (NCBI GEO GSE96920; Soh et al., 2017) were re-analyzed, with additional details provided in the supplementary Materials and Methods. YTHDC2-bound mRNAs identified via CLIP-seq in P8 and P10 testes were extracted from Saito et al. (2022) (Tables S1 and S2: http://genesdev.cshlp.org/content/suppl/2022/01/19/gad.349190.121.DC1/Supplemental_Tables.xlsx). RBM46 eCLIP data from P12-P14 testes (NCBI GEO GSE197282) (Qian et al., 2022) were re-analyzed, with additional details provided in the supplementary Materials and Methods. See supplementary Materials and Methods for details on target comparison to MEIOC scRNA-seq and bulk RNA-seq datasets.

### Identification of transcriptional repressors that are inhibited by MEIOC-YTHDC2-RBM46

To identify transcriptional repressors that are inhibited by MEIOC-YTHDC2-RBM46, we first identified transcripts that were (1) downregulated by MEIOC in the In/B, B G2M and/or pL G1 clusters; and (2) targeted by MEIOC-YTHDC2-RBM46. We then manually examined the ‘Function’ description of the UniProtKB database (release 2020_01; www.uniprot.org) for these mouse proteins. Any proteins that were annotated as repressing transcription or gene expression were considered to be transcriptional repressors.

### Re-analysis of E2F6 and MGA ChIP-seq datasets and RNA-seq datasets from ESCs

The following datasets were re-analyzed here: E2F6 ChIP-seq and input data from wild-type and *E2f6* knockout mouse ESCs (NCBI GEO GSE149025) (Dahlet et al., 2021); MGA and IgG ChIP-seq from wild-type mouse ESCs (ArrayExpress E-MTAB-6007) (Stielow et al., 2018); mouse ESC RNA-seq data from wild-type and *E2f6* knockout samples (NCBI GEO GSE149025) (Dahlet et al., 2021); and wild-type and *Mga* knockout samples (NCBI GEO GSE144141) (Qin et al., 2021). See supplementary Materials and Methods for further details.

### Immunostaining

Immunostaining was carried out in tissue sections using the following primary antibodies: anti- DMRT1 (Santa Cruz Biotechnology sc-377167, 1:200 dilution for fluorescent staining); anti-MEIOSIN (guinea pig polyclonal from Ishiguro et al., 2020, 1:100 dilution for fluorescent staining); anti-STRA8 (Abcam ab49405, 1:500 dilution for chromogenic staining or 1:200 for fluorescent staining); and anti-STRA8 (Abcam ab49602, 1:200 dilution for fluorescent staining). See supplementary Materials and Methods for further details.

## Supplementary Material



10.1242/develop.202740_sup1 Supplementary information

Table S1. Supplementary table and stats by figure panel

Table S2. scRNA-seq analysis of WT germ cell clusters from P15 testis

Table S3. scRNA-seq differential expression analysis (DEA)

Table S4. scRNA-seq DEA WT v. KO Gene Ontology analysis

Table S5. Associated cell cycle phase of MEIOC-downregulated genes

Table S6. MEIOC, YTHDC2, and RBM46 target analysis

Table S7. mRNA stability and transcriptional rate from bulk RNA-seq data

Table S8. Number of cells per scRNA-seq cluster in P15 testes

Table S9. bulk RNA-seq differential expression analysis, WT vs. *Meioc* KO

Table S10. Cell cycle analysis of bulk RNA-seq *Meioc* and *Stra8* datasets

Table S11. bulk RNA-seq Gene Ontology analysis

Table S12. Reanalysis of E2f6 and Mga data from embryonic stem cells

Table S13. MEIOSIN quantification in WT v Meioc KO
