# Supplementary material for: Destabilization of mRNAs enhances competence to initiate meiosis in mouse spermatogenic cells
Source: Development. 2024 Jul 15;151(14):dev202740. doi: 10.1242/dev.202740 (PMC11273298; doi:10.1242/dev.202740)
Supplement: Supplementary file 1 [file develop-151-202740_review_history.pdf]

Destabilization of mRNAs enhances competence to initiate meiosis in mouse spermatogenic cells

Natalie G. Pfaltzgraff, Bingrun Liu, Dirk G. de Rooij, David C. Page and Maria Mikedis
DOI: 10.1242/dev.202740

Editor: Haruhiko Koseki

Review timeline

Original submission:	29 January 2024
Editorial decision:	28 February 2024
First revision received:	18 April 2024
Editorial decision:	17 May 2024
Second revision received:	2 June 2024
Accepted:	7 June 2024

Original submission

First decision letter

MS ID#: DEVELOP/2024/202740

MS TITLE: Destabilization of mRNAs enhances competence to initiate meiosis in mouse spermatogenic cells

AUTHORS: Maria Mikedis, Natalie G. Pfaltzgraff, Bingrun Liu, Dirk G. de Rooij, and David C. Page

I have now received all the referees' reports on the above manuscript, and have reached a decision. The referees' comments are appended below, or you can access them online: please go to BenchPress and click on the 'Manuscripts with Decisions' queue in the Author Area.

As you will see, the referees express considerable interest in your work, but have some significant criticisms and recommend a substantial revision of your manuscript before we can consider publication. If you are able to revise the manuscript along the lines suggested, which may involve further experiments, I will be happy to receive a revised version of the manuscript. Your revised paper will be re-reviewed by one or more of the original referees, and acceptance of your manuscript will depend on your addressing satisfactorily the reviewers' major concerns. Please also note that Development will normally permit only one round of major revision. If it would be helpful, you are welcome to contact us to discuss your revision in greater detail. Please send us a point-by-point response indicating your plans for addressing the referees' comments, and we will look over this and provide further guidance.

Please attend to all of the reviewers' comments and ensure that you clearly highlight all changes made in the revised manuscript. Please avoid using 'Tracked changes' in Word files as these are lost in PDF conversion. I should be grateful if you would also provide a point-by-point response detailing how you have dealt with the points raised by the reviewers in the 'Response to Reviewers' box. If you do not agree with any of their criticisms or suggestions please explain clearly why this is so.

Reviewer 1

Advance summary and potential significance to field

© 2024. Published by The Company of Biologists under the terms of the Creative Commons Attribution License (https://creativecommons.org/licenses/by/4.0/).

The Mikedes et al., proposed the gene regulatory network that controls the successful transcriptomic transition from mitosis to meiosis in spermatogenesis. Based on single cell-RNA-seq data and publicly available NGS datasets, the authors found that the post-transcriptional regulatory complex, the MEIOC-YTHDC2-RBM46 complex acts as RNA destabilizer from an earlier stage in spermatogenesis than previously thought to make germ cells competent for meiosis. The data is comprehensive and of technical high quality.

Main message through the manuscript is clear. Overall manuscript is well organized, logical and raises several insights into mitosis-to-meiosis transition. This work also has major implications for the evolution of the mitosis-meiosis transition. Therefore, the manuscript should be open for the field. However, this reviewer raised several concerns as described below.

Comments for the author

Comments
Figure 1: Although analysis of NGS data has been well done, in this reviewer's impression, the overall description of the phenotypes of Meioc-KO is somewhat missing in this article. It would be better to strengthen their idea if the authors carefully describe more about the phenotypes of Meioc-KO based on their scRNA-seq. The gene expression changes at an earlier differentiation have been well described by the authors in this article. However, as they have shown in Figure 1, after pL G1 cells, Meioc KO cells follow a completely different cell differentiation trajectory. What is happening in these cells in terms of gene expression programs or cell characteristics? To help the readers to understand exactly what is happening in Meioc-KO, please specify the gene expression differences at a later stage.
Otherwise, the readers would be hard to follow the events that happen in the absence of MEIOC from the first step to final outcome.

Line104-108 : The author simply stated that MEIOC activity is associated with transcriptomic changes at pleleptotene, and drives a major transcriptomic shift in spermatogenic cells at the meiotic G1/S transition.
The authors should state more comprehensively and clearly results and interpretation of bulk RNA-seq data

Lines 141-143; Based on the gene enrichment analysis, the authors concluded that MEIOC prevents the occurrence of an aberrant M phase during meiotic prophase I. If this is one of the critical functions of MEIOC in spermatogenesis, the accumulation of aberrant M phase of cells should be observed. Please show the quantified data on this point. These data will help the readers to understand the outcome of Meioc deficiency in testis.

Lines 175-191; Please discuss on the differences between MEIOC-YTHDC2-RBM46 targets and MEIOC-RBM46 targets. What are their characteristics? Do the authors think there is a biological significance? If so, please discuss function of MEIOC in earlier stage and later stage of spermatogenesis.

As the authors have shown in Figure 3D, MEIOC suppresses Mga in In/B spermatogonia. This raises the possibility that in the Meioc-KO, cell differentiation is also affected due to the prolonged expression of MGA. Please discuss on this point based on the authors' data.

The authors proposed that E2F6-MGA, components of PRC1.6, as primary targets of the MEIOC-YTHDC2-RMB46 complex. If the suppression of E2F6/MGA by MEIOC has a critical role in spermatogenesis and meiotic entry, the downregulation of MGA should be observed in the spermatogonial population. Could the authors show the other supporting data such as IF etc on the relationship between MEIOC and E2F6/MGA?
Such data would support the authors' conclusion and make it more robust.

The term "MEIOC-dependent genes" is confusing as the authors also use "MEIOC-upregulatd genes" and "MEIOC-downregulatd genes" in the text. Please state clearly the definition of "MEIOC-dependent genes".
This is also applied to "STRA8-dependent genes".

© 2024. Published by The Company of Biologists under the terms of the Creative Commons Attribution License (https://creativecommons.org/licenses/by/4.0/).

Line 322 : The authors stated that ~60-70% of MEIOC-upregulated genes are directly activated by STRA8-MEIOSIN. They also claimed that genes repressed by both E2F6 and MGA were overrepresented among MEIOC-upregulated transcripts beginning in the mitotic In/B clusters and continuing through the mitosis-to-meiosis transition (Fig. 3E) and STRA8-activated genes are repressed by E2F6 and/or MGA (Fig. S12E). This reviewer wonders whether both STRA8/MEIOSIN- and E2F6-binding motives are found in the promoters of MEIOC-upregulated genes. Please clarify this issue.

A similar single cell transcriptome analysis at the mitosis-to-meiosis transition in female germ cells was reported (Shimada et al, Nat Commun 2023). It would be more informative to add discussion and/or introduction on what is known or unknown regarding at the meiotic G1/S transition in males and females and how the authors' finding in male germ cells are relevant to the previous studies on oogonia.

Reviewer 2

Advance summary and potential significance to field

This manuscript describes new roles of interaction between MEIOC and YTHDC2 and RBM46. The authors used single cell and bulk RNA sequencing to dissect their roles. Although all of these molecules are known to be involved in meiosis progression, the analyses in this manuscript bring a new light to understand their roles.

Comments for the author

Unfortunately, analyses were performed mostly by RNA levels, which make me wonder if these interactions are really significant at the protein level.

1. Confirmation of protein expression In Fig. 1 and Fig. 2, the expression analysis suggested that Meioc KO mice contain limited number of cells. However, I was not sure if these mRNA expression are really reflecting protein expression. I think that the authors are assuming that the KO cells are expressing the target mRNA at the same level as the wild-type cells, which may or may not be the case. Confirmation of protein expression in Meioc KO mice would strengthen the manuscript. Analysis of cell cycle proteins is needed if the authors want to conclude on the disturbed cell cycle. Immunohistochemical analysis of YTHDC2, RBM46, E2F6, MGA, STRA8 and MEIOSIN in Meioc KO mice also confirms the result of single cell analysis.

2. Confirmation of cell population Almost all figures in the manuscript are based on RNA analysis, I could not tell how the cell populations are different between Meioc KO and wild-type mice. This can be done by conventional histological analysis and immunohistochemisry. It would be useful for readers to compare whether the changes in gene expression is reflecting the difference in cell population or gene expression levels. Otherwise, I cannot tell whether accumulation of meiotic cell transcripts occurred in spermatogonia or spermatocytes.

3. Expression of MEIOC-RBM46-YTHDC2 The authors showed collaboration of the three proteins. If so, they should show the same protein localization during spermatogenesis. What stage are they localized together by immunohistochemistry? What happens to this complex in Meioc KO mice?

4. Impact of E2F6 and MGA on meiotic gene expression Analysis of ES cell transcript does not seem to be appropriate to conclude their effect because this is a different cell type. I think that this has to be confirmed by in vivo analysis by gene KD and overexpression.

5. Target of SCF complex The authors suggest that SCF complex degrades DMRT1 and promotes meiosis. How can the authors exclude the possibility that the SCF complex has additional targets? Because the previous report used ubiquitous promoter (CAG) to overexpress Dmrt1 (Nakagawa et al., Development), I am not sure if Dmrt1 expression in Sertoli cells are responsible for the observed phenotype.

© 2024. Published by The Company of Biologists under the terms of the Creative Commons Attribution License (https://creativecommons.org/licenses/by/4.0/).

Reviewer 3

Advance summary and potential significance to field

This manuscript deals with the mechanism of MEIOC in meiotic initiation in mouse. MEIOC is known to form a complex with RNA-binding proteins YTHDC2 and RBM46. This protein complex is required for mitotic to meiotic transition. scRNA-seq and bulk RNA-seq of synchronized wild type and MEOIC-null preleptotene-enriched testes were performed. These new datasets were analyzed with other RNA-seq, RIP-seq, CLIP-seq, ChIP-seq data of various relevant proteins (MEIOC, YTHDC2, RBM46, STRA8, MEIOSIN, E2F6, and MGA). The analyses are comprehensive and carefully controlled. They found that MEIOIC modulates transcriptome in spermatogonia before meiotic initiation, which was earlier than previously believed. This study confirmed that MEIOIC destabilizes transcripts through partnering with YTHDC2 and RBM46. However, more transcripts were up-regulated in MEIOC-null germ cells. They showed that MEIOIC destabilizes transcriptional repressors E2F6 and MGA in spermatogonia and thus de-represses expression of many meiosis genes including MEIOSIN indirectly. The scRNA-seq analysis is very informative in defining the spermatogonia and preleptotene cell clusters. The conclusions are supported by the data. This study provides novel and significant mechanistic insights into the role of the MEIOC-YTHDC2-RBM46 complex in meiotic initiation. I support its publication in Development.

Comments for the author

I have only minor comments:

1) Introduction: In the second paragraph, nutrient restriction needs to be briefly described as it synergizes with retinoic acid in meiosis induction in mouse spermatogonia (Zhang et al. 2021; PMID: 33741948).

2) Results (L264-266): The concluding sentences are a bit awkward and redundant “We conclude that MEIOC’s repression of E2F6 and MGA does not lead to downregulation of gene expression. Instead, it derepresses gene expression.” Delete the second sentence (redundant). Suggestion: “We conclude that MEIOC’s repression of E2F6 and MGA leads to upregulation of gene expression”.

3) Discussion (L381): Please also cite this study (Li et al. 2022; PMID: 35305312). This publication reached the same conclusions as Saito et al. 2022 study.

First revision

Author response to reviewers' comments

We thank the reviewers for their time, as their thorough readings and thoughtful comments have helped us to greatly improve the manuscript’s analyses and clarity. In response to their comments, we have added new analyses and made numerous revisions to text and figures. We detail our revisions in response to the comments below.

Reviewer 1
The Mikedis et al., proposed the gene regulatory network that controls the successful transcriptomic transition from mitosis to meiosis in spermatogenesis. Based on single cell-RNA-seq data and publicly available NGS datasets, the authors found that the post-transcriptional regulatory complex, the MEIOC-YTHDC2-RBM46 complex acts as RNA destabilizer from an earlier stage in spermatogenesis than previously thought to make germ cells competent for meiosis. The data is comprehensive and of technical high quality. Main message through the manuscript is clear. Overall manuscript is well organized, logical and raises several insights into mitosis-to-meiosis transition. This work also has major implications for the evolution of the mitosis-meiosis transition. Therefore, the manuscript should be open for the field. However, this reviewer raised

© 2024. Published by The Company of Biologists under the terms of the Creative Commons Attribution License (https://creativecommons.org/licenses/by/4.0/).

several concerns as described below.

Figure 1: Although analysis of NGS data has been well done, in this reviewer's impression, the overall description of the phenotypes of Meioc-KO is somewhat missing in this article. It would be better to strengthen their idea if the authors carefully describe more about the phenotypes of Meioc-KO based on their scRNA-seq. The gene expression changes at an earlier differentiation have been well described by the authors in this article. However, as they have shown in Figure 1, after pL G1 cells, Meioc KO cells follow a completely different cell differentiation trajectory. What is happening in these cells in terms of gene expression programs or cell characteristics? To help the readers to understand exactly what is happening not in Meioc-KO, please specify the gene expression differences at a later stage. Otherwise, the readers would be hard to follow the events that happen in the absence of MEIOC from the first step to final outcome.

> We have added text to clarify the gene expression changes observed after the pL G1 cluster as well as how these changes correspond to the histologically defined phenotype of the Meioc KO:
>
> Lines 107-110
> Lines 134-136
> Lines 169-171
> Lines 176-181
> Lines 185-188

Line104-108 : The author simply stated that MEIOC activity is associated with transcriptomic changes at preleptotene, and drives a major transcriptomic shift in spermatogenic cells at the meiotic G1/S transition. The authors should state more comprehensively and clearly results and interpretation of bulk RNA-seq data.

> We have added a more comprehensive description of how the bulk RNA-seq data supports the scRNA-seq analysis in demonstrating a transcriptomic shift in spermatogenic cells at the meiotic G1/S transition (lines 120-133).

Lines 141-143; Based on the gene enrichment analysis, the authors concluded that MEIOC prevents the occurrence of an aberrant M phase during meiotic prophase I. If this is one of the critical functions of MEIOC in spermatogenesis, the accumulation of aberrant M phase of cells should be observed. Please show the quantified data on this point. These data will help the readers to understand the outcome of Meioc deficiency in testis.

> We believe the "Mut" cluster found in the *Meioc* KO, but not wild-type, testes represents the aberrant M phase cells histologically identified in the Meioc KO testes. We have clarified this in the text (lines 111-115), and we have added figure panels to show the percentage of these Mut cells as well as their cell cycle phase (Fig. S1C,D; Table S8).

Lines 175-191; Please discuss on the differences between MEIOC-YTHDC2-RBM46 targets and MEIOC-RBM46 targets. What are their characteristics? Do the authors think there is a biological significance? If so, please discuss function of MEIOC in earlier stage and later stage of spermatogenesis.

> We have added GO analysis of MEIOC-YTHDC2-RBM46 targets and MEIOC-RBM46 targets. (Table S6, tabs "4. GO analysis MYR targets" and "5. GO analysis MR-only targets"). We have also modified the text to note that MEIOC-YTHDC2-RBM46 targets were enriched for GO annotations associated with RNA stability, while MEIOC-RBM46- only targets did not exhibit statistically significant enrichment (lines 219-221).
>
> It is unclear to the authors if activity of MEIOC-RBM46 (independent of YTHDC2) is biologically relevant, as we now state directly (lines 232-235).

As the authors have shown in Figure 3D, MEIOC suppresses Mga in In/B spermatogonia. This raises the possibility that in the Meioc-KO, cell differentiation is also affected due to the prolonged expression of MGA. Please discuss on this point based on the authors' data.

> We agree that prolonged expression of MGA in the Meioc KO may affect cell differentiation. Indeed, we find that genes repressed by E2F6 and MGA are enriched among MEIOC-upregulated genes in In/B spermatogonia. At this stage, *Mga* is downregulated while *E2f6* is unaffected. Therefore, MEIOC's destabilization of *Mga* mRNA likely impacts gene expression and cell differentiation in In/B spermatogonia. We have

© 2024. Published by The Company of Biologists under the terms of the Creative Commons Attribution License (https://creativecommons.org/licenses/by/4.0/).

clarified these points in the text (lines 302-305; lines 475-476).

The authors proposed that E2F6-MGA, components of PRC1.6, as primary targets of the MEIOC-YTHDC2-RMB46 complex. If the suppression of E2F6/MGA by MEIOC has a critical role in spermatogenesis and meiotic entry, the downregulation of MGA should be observed in the spermatogonial population. Could the authors show the other supporting data such as IF etc on the relationship between MEIOC and E2F6/MGA? Such data would support the authors' conclusion and make it more robust.

We agree that IF data showing changes in E2F6 and MGA protein expression would support our conclusions. We have attempted these experiments by testing multiple antibodies for immunohistochemistry in the testis. However, we are unconvinced that the antibodies are sufficiently specific and therefore have not added these experiments to the manuscript.

Our bioinformatic analyses support the conclusion that MEIOC's destabilization of *E2f6* and *Mga* transcripts ultimately affects E2F6 and MGA protein function, as the MEIOC- dependent program exhibits a transcriptomic signature consistent with altered E2F6- MGA protein activity (lines 300-320, 424-442; Fig. 3E, 4E).

The term "MEIOC-dependent genes" is confusing as the authors also use "MEIOC-upregulatd genes" and "MEIOC-downregulatd genes" in the text. Please state clearly the definition of "MEIOC-dependent genes". This is also applied to "STRA8-dependent genes".

We now explicitly define "MEIOC-dependent genes" as "MEIOC-upregulated or downregulated genes" (lines 202-203). We also define "STRA8-dependent genes" (lines 378-379).

Line 322 : The authors stated that ~60-70% of MEIOC-upregulated genes are directly activated by STRA8-MEIOSIN. They also claimed that genes repressed by both E2F6 and MGA were overrepresented among MEIOC-upregulated transcripts beginning in the mitotic In/B clusters and continuing through the mitosis-to-meiosis transition (Fig. 3E) and STRA8-activated genes are repressed by E2F6 and/or MGA (Fig. S12E). This reviewer wonders whether both STRA8/MEIOSIN- and E2F6-binding motives are found in the promoters of MEIOC- upregulated genes. Please clarify this issue.

For the bulk RNA-seq analysis, we show that the top motif enriched in MEIOC-upregulated genes matches the STRA8-MEIOSIN motif in Fig. S12D. We now include the second ranked enriched motif, which matches E2F6's binding motif, in this figure panel. We have added text to highlight this second ranked motif (lines 405-406).

For the scRNA-seq analysis, we now state that de novo motif analysis failed to identify enrichment of any motif (lines 399-401). Given that experimentally identified STRA8-activated genes were enriched among MEIOC-upregulated genes from the scRNA-seq analysis (Fig. 4D), we attribute the absence of motif enrichment to the sparsity of scRNA-seq data.

A similar single cell transcriptome analysis at the mitosis-to-meiosis transition in female germ cells was reported (Shimada et al, Nat Commun 2023). It would be more informative to add discussion and/or introduction on what is known or unknown regarding at the meiotic G1/S transition in males and females, and how the authors' finding in male germ cells are relevant to the previous studies on oogonia.

In the discussion, we now discuss the mitosis-to-meiosis transition in oogonia vs spermatogenic cells (lines 481-491).

Reviewer 2

This manuscript describes new roles of interaction between MEIOC and YTHDC2 and RBM46. The authors used single cell and bulk RNA sequencing to dissect their roles. Although all of these molecules are known to be involved in meiosis progression, the analyses in this manuscript bring a new light to understand their roles.

Unfortunately, analyses were performed mostly by RNA levels, which make me wonder if these interactions are really significant at the protein level.

© 2024. Published by The Company of Biologists under the terms of the Creative Commons Attribution License (https://creativecommons.org/licenses/by/4.0/).

1. Confirmation of protein expression

In Fig. 1 and Fig. 2, the expression analysis suggested that Meioc KO mice contain limited number of cells. However, I was not sure if these mRNA expression are really reflecting protein expression. I think that the authors are assuming that the KO cells are expressing the target mRNA at the same level as the wild-type cells, which may or may not be the case. Confirmation of protein expression in Meioc KO mice would strengthen the manuscript. Analysis of cell cycle proteins is needed if the authors want to conclude on the disturbed cell cycle. Immunohistochemical analysis of YTHDC2, RBM46, E2F6, MGA, STRA8 and MEIOSIN in Meioc KO mice also confirms the result of single cell analysis.

We appreciate the point that mRNA expression does not necessarily reflect protein expression. To this end, we have verified that Meioc KO preleptotene spermatocytes exhibit reduced MEIOSIN protein levels, based on immunofluorescent signal (Fig. S10A; lines 336-337).

We have added text to note that we and others detect STRA8 protein expression in Meioc KO spermatogenic cells at the mitosis-to-meiosis transition (Fig. S10A; lines 372-374).

We have added text to note that YTHDC2 protein continues to be expressed in Meioc KO spermatogenic cells, based on prior studies (lines 508-510).

We have attempted RBM46, E2F6, and MGA experiments by testing multiple antibodies for immunohistochemistry in the testis. However, we are unconvinced of antibody specificity and therefore have not added these experiments to the manuscript. As noted in our response to Reviewer 1, our bioinformatic analyses provide supporting evidence that MEIOC ultimately impacts E2F6-MGA protein function (lines 300-320, 424-442; Fig. 3E, 4E).

The disrupted cell cycle is evidenced by Meioc KO spermatocytes' premature progression to an aberrant metaphase state during meiotic prophase I and their prolonged expression of mitotic S and G2/M Cyclin A2, when stage-matched wild-type spermatocytes have lost their Cyclin A2 protein expression (Abby et al., 2016; Soh et al., 2017). We now describe this evidence in the text (lines 179-181; 185-188).

2. Confirmation of cell population

Almost all figures in the manuscript are based on RNA analysis, I could not tell how the cell populations are different between Meioc KO and wild-type mice. This can be done by conventional histological analysis and immunohistochemistry. It would be useful for readers to compare whether the changes in gene expression is reflecting the difference in cell population or gene expression levels. Otherwise, I cannot tell whether accumulation of meiotic cell transcripts occurred in spermatogonia or spermatocytes.

As noted in our response to Reviewer 1, we have added additional text to correlate observed gene expression changes from our study to the histologically defined phenotype of the Meioc KO:

Lines 107-110
Lines 134-136
Lines 169-171
Lines 176-181
Lines 185-188

Prior studies reported that *Meioc*-null spermatogenic cells diverge from wild-type controls after entering meiotic S phase at the preleptotene stage (Abby et al., 2016; Soh et al., 2017) (lines 134-136). As no immunohistological differences have been detected in Meioc KO spermatogonia, the MEIOC-dependent transcriptomic changes identified in spermatogonial clusters reflect changes in transcript abundance and not differences in cell population.

3. Expression of MEIOC-RBM46-YTHDC2

The authors showed collaboration of the three proteins. If so, they should show the same protein localization during spermatogenesis. What stage are they localized together by immunohistochemistry? What happens to this complex in Meioc KO mice?

© 2024. Published by The Company of Biologists under the terms of the Creative Commons Attribution License (https://creativecommons.org/licenses/by/4.0/).

We have added text describing the previously reported localization patterns of MEIOC, YTHDC2, and RBM46 proteins (lines 140-144).

YTHDC2 is expressed normally in the Meioc KO (Abby et al., 2016; Soh et al., 2017). It is not known whether loss of MEIOC affects RBM46 protein expression, as we do not have a working RBM46 antibody for immunostaining. (Published RBM46 immunostaining experiments used anti-FLAG to detect FLAG-tagged RBM46 (Peart et al., 2022; Qian et al., 2022)). We have made corresponding changes to the text (lines 508- 510).

4. Impact of E2F6 and MGA on meiotic gene expression
Analysis of ES cell transcript does not seem to be appropriate to conclude their effect because this is a different cell type. I think that this has to be confirmed by in vivo analysis by gene KD and overexpression.

ES cells are an appropriate model system for how E2F6 and MGA may impact meiotic entry because ES cells can be induced to enter meiosis via genetic ablation of *Max*, which encodes MGA's binding partner and a PRC1.6 subunit (Suzuki et al., 2016). We have clarified this in the text (lines 296-298).

We are actively pursuing in vivo studies of MGA and E2F6 function during spermatogenesis. However, these experiments require extensive work in additional mouse lines and are therefore beyond the scope of the current manuscript.

5. Target of SCF complex
The authors suggest that SCF complex degrades DMRT1 and promotes meiosis. How can the authors exclude the possibility that the SCF complex has additional targets? Because the previous report used ubiquitous promoter (CAG) to overexpress Dmrt1 (Nakagawa et al., Development), I am not sure if Dmrt1 expression in Sertoli cells are responsible for the observed phenotype.

The manuscript's focus is on our newfound understanding of MEIOC's role in meiotic initiation. We cite the published model on the SCF complex, DMRT1, and meiosis in order to place our MEIOC model with the context of the current literature.

To clarify, DMRT1 protein is expressed in spermatogenic cells (in addition to Sertoli cells). Nakagawa et al's model that the SCF complex degrades DMRT1 is based on:

(i) In a β-TrCP1/2 double conditional knockout mouse model, DMRT1 protein accumulates in spermatogenic cells;
(ii) in 293T cells with overexpressed β-TrCP1 and β-TrCP2, DMRT1 exhibits increased ubiquitination. This ubiquitination is not observed for a modified DMRT1 with a mutated degron motif, to which the SCF complex binds
(iii) in mouse embryonic fibroblasts with Cre-mediated reduction of β-TrCP1 and β-TrCP2, DMRT1 protein accumulates
(iv) in a Cre-inducible mouse model that expresses a bicistronic *Dmrt1* and *Gfp* mRNA from a CAG promoter, DMRT1 and GFP are co-expressed in mitotic spermatogenic cells, but only GFP and not DMRT1 are expressed in spermatogenic cells at the mitosis-to-meiosis transition. As both proteins are translated from the same mRNA, and *Dmrt1* is translated first, this suggests that DMRT1 protein is repressed after translation in spermatogenic cells at the mitosis-to-meiosis transition.

We cannot exclude that possibility that the SCF complex has additional targets, but we are unaware of any such evidence to date.

Reviewer 3
This manuscript deals with the mechanism of MEIOC in meiotic initiation in mouse. MEIOC is known to form a complex with RNA-binding proteins YTHDC2 and RBM46. This protein complex is required for mitotic to meiotic transition. scRNAseq and bulk RNA-seq of synchronized wild type and MEOIC-null preleptotene-enriched testes were performed. These new datasets were analyzed with other RNA-seq, RIP-seq, CLIP-seq, ChIP-seq data of various relevant proteins (MEIOC, YTHDC2, RBM46, STRA8, MEIOSIN, E2F6, and MGA). The analyses are comprehensive and carefully controlled. They found that MEIOIC modulates transcriptome in spermatogonia before meiotic initiation, which was earlier than previously believed. This study confirmed that MEIOIC

© 2024. Published by The Company of Biologists under the terms of the Creative Commons Attribution License (https://creativecommons.org/licenses/by/4.0/).

destabilizes transcripts through partnering with YTHDC2 and RBM46. However, more transcripts were up-regulated in MEIOC-null germ cells. They showed that MEIOIC destabilizes transcriptional repressors E2F6 and MGA in spermatogonia and thus de-represses expression of many meiosis genes including MEIOSIN indirectly. The scRNA-seq analysis is very informative in defining the spermatogonia and preleptotene cell clusters. The conclusions are supported by the data. This study provides novel and significant mechanistic insights into the role of the MEIOC-YTHDC2-RBM46 complex in meiotic initiation. I support its publication in Development.

I have only minor comments:
1) Introduction: In the second paragraph, nutrient restriction needs to be briefly described as it synergizes with retinoic acid in meiosis induction in mouse spermatogonia (Zhang et al. 2021; PMID: 33741948).

> We now describe the role of nutrient restriction in meiotic induction, citing Zhang et al., 2021 (lines 40-42).

2) Results (L264-266): The concluding sentences are a bit awkward and redundant "We conclude that MEIOC's repression of E2F6 and MGA does not lead to downregulation of gene expression. Instead, it derepresses gene expression." Delete the second sentence (redundant). Suggestion: "We conclude that MEIOC's repression of E2F6 and MGA leads to upregulation of gene expression".

> We have changed the text as suggested (lines 319-320)

3) Discussion (L381): Please also cite this study (Li et al. 2022; PMID: 35305312). This publication reached the same conclusions as Saito et al. 2022 study.

> Saito et al., 2022 carried out ribosome profiling in wild-type and Ythdc2 KO testes. These experiments failed to detect YTHDC2-dependent changes in translational efficiency. Li et al did not measure translational levels in the Ythdc2 KO. For greater clarity, we have modified the text as follows (yellow indicates modified text):
>
> "a recent study using ribosome profiling found that YTHDC2 does not affect translation in postnatal testes (Saito et al., 2022)." lines 466

Second decision letter

MS ID#: DEVELOP/2024/202740

MS TITLE: Destabilization of mRNAs enhances competence to initiate meiosis in mouse spermatogenic cells

AUTHORS: Natalie G. Pfaltzgraff, Bingrun Liu, Dirk G. de Rooij, David C. Page, and Maria Mikedis

I have now received all the referees' reports on the above manuscript, and have reached a decision. The referees' comments are appended below, or you can access them online: please go to BenchPress and click on the 'Manuscripts with Decisions' queue in the Author Area.

As you will see, the referees express considerable interest in your work, but the Reviewer 2 still expressed significant concerns for protein expression levels of genes of interests. Based on this comment, we recommend a further revision of your manuscript before we can consider publication. If you are able to revise the manuscript along the lines suggested, which may involve further experiments, I will be happy receive a revised version of the manuscript. Your revised paper will be re-reviewed by one or more of the original referees, and acceptance of your manuscript will depend on your addressing satisfactorily the reviewers' major concerns. Please also note that Development will normally permit only one round of major revision. If it would be helpful, you are welcome to contact us to discuss your revision in greater detail. Please send us a point-by-point response indicating your plans for addressing the referees' comments, and we will look over this and provide further guidance.

© 2024. Published by The Company of Biologists under the terms of the Creative Commons Attribution License (https://creativecommons.org/licenses/by/4.0/).

Please attend to all of the reviewers' comments and ensure that you clearly highlight all changes made in the revised manuscript. Please avoid using 'Tracked changes' in Word files as these are lost in PDF conversion. I should be grateful if you would also provide a point-by-point response detailing how you have dealt with the points raised by the reviewers in the 'Response to Reviewers' box. If you do not agree with any of their criticisms or suggestions please explain clearly why this is so.

Reviewer 1

Advance summary and potential significance to field

Now revised manuscript satisfied most of my concerns. Since their scRNA-seq analysis is well informed in defining the states of spermatogonia and preleptotene spermatocytes(in/B spermatogonia, pLep G1, pLep early S, pLep late S), this study provides previously unforeseen mechanistic insights into the role of the destabilization of pre-meiotic transcripts by MEIOC in mitosis – meiosis transition. I support its publication in Development. Kei-ichiro Ishiguro, Ph.D.

Comments for the author

Now revised manuscript satisfied most of my concerns. Since their scRNA-seq analysis is well informed in defining the states of spermatogonia and preleptotene spermatocytes(in/B spermatogonia, pLep G1, pLep early S, pLep late S), this study provides previously unforeseen mechanistic insights into the role of the destabilization of pre-meiotic transcripts by MEIOC in mitosis – meiosis transition. I support its publication in Development. Kei-ichiro Ishiguro, Ph.D.

Reviewer 2

Advance summary and potential significance to field

This manuscript describes new roles of interaction between MEIOC and YTHDC2 and RBM46. The authors used single cell and bulk RNA sequencing to dissect their roles. Although all of these molecules are known to be involved in meiosis progression, the analyses in this manuscript bring a new light to understand their roles.

Comments for the author

Unfortunately, the authors did not show protein expression. The authors quantified only MEIOSIN by immunostaining. I am not sure if the conclusion from mRNA data is valid without protein expression. There are many genes in spermatogenesis that are not translated into proteins.
The authors did not sufficiently address my concern. There are many assumptions in the manuscript, which I am not able to follow.

Reviewer 3

Advance summary and potential significance to field

My concerns have been addressed.

Comments for the author

No more concerns.

Second revision

Author response to reviewers' comments

© 2024. Published by The Company of Biologists under the terms of the Creative Commons Attribution License (https://creativecommons.org/licenses/by/4.0/).

We thank the reviewers for helping us improve the manuscript. In addition to a formal review, we received a further comment from Reviewer 2 communicated through our email correspondence with the handling editor. Below, we provide our responses to these comments and note where text revisions have been made.

Reviewer 2 comments in black; authors response in blue:

From formal review:
Unfortunately, the authors did not show protein expression. The authors quantified only MEIOSIN by immunostaining. I am not sure if the conclusion from mRNA data is valid without protein expression. There are many genes in spermatogenesis that are not translated into proteins.

> Abby et al., (2016) and Soh et al. (2017) demonstrated that STRA8 and YTHDC2 are still expressed in the *Meioc* KO, using immunostaining and western blotting.
>
> We have not been able to identify specific antibodies for immunochemical analysis of RBM46, E2F6, or MGA. We tested multiple antibodies without success. *E2f6*, *Mga*, and *Rbm46* transcripts are actively translated into protein during the transition from mitosis to meiosis, based on ribosome profiling data from Saito et al., 2022. In support of our model that MEIOC represses *E2f6* and *Mga* mRNA, we bioinformatically identified transcriptomic changes in the *Meioc* KO that would be predicted to occur if E2F6-MGA protein is elevated (Fig. 3E, 4E).

From additional correspondence with handling editor:
Do MEIOC, YTHDC2 and RBM46 really form complexes in spermatocytes or earlier?

> Abby et al. (2016) and Soh et al. (2017) concluded that MEIOC and YTHDC2 act as a complex based on co-immunoprecipitation. Li et al. (2022) and Qian et al. (2022) concluded that RBM46 acts in complex with MEIOC and YTHDC2 based on co-immunoprecipitation.
>
> These conclusions are now summarized in the manuscript as lines 75-76 and 214-215.

Third decision letter

MS ID#: DEVELOP/2024/202740

MS TITLE: Destabilization of mRNAs enhances competence to initiate meiosis in mouse spermatogenic cells

AUTHORS: Natalie G. Pfaltzgraff, Bingrun Liu, Dirk G. de Rooij, David C. Page, and Maria Mikedis
ARTICLE TYPE: Research Article

I am happy to tell you that your manuscript has been accepted for publication in Development, pending our standard ethics checks.

© 2024. Published by The Company of Biologists under the terms of the Creative Commons Attribution License (https://creativecommons.org/licenses/by/4.0/).
